# Spike avalanches *in vivo* suggest a driven, slightly subcritical brain state

**DOI:** 10.3389/fnsys.2014.00108

**Published:** 2014-06-24

**Authors:** Viola Priesemann, Michael Wibral, Mario Valderrama, Robert Pröpper, Michel Le Van Quyen, Theo Geisel, Jochen Triesch, Danko Nikolić, Matthias H. J. Munk

**Affiliations:** ^1^Department of Non-linear Dynamics, Max Planck Institute for Dynamics and Self-OrganizationGöttingen, Germany; ^2^Bernstein Center for Computational NeuroscienceGöttingen, Germany; ^3^Frankfurt Institute for Advanced StudiesFrankfurt, Germany; ^4^Department of Neurophysiology, Max Planck Institute for Brain ResearchFrankfurt, Germany; ^5^Magnetoencephalography Unit, Brain Imaging Center, Johann Wolfgang Goethe UniversityFrankfurt, Germany; ^6^Ernst Strüngmann Institute for Neuroscience in Cooperation with Max Planck SocietyFrankfurt, Germany; ^7^Department of Biomedical Engineering, University of Los AndesBogotá, Colombia; ^8^Neural Information Processing Group, Department of Software Engineering and Theoretical Computer Science, TU BerlinBerlin, Germany; ^9^Bernstein Center for Computational NeuroscienceBerlin, Germany; ^10^Centre de Recherche de l’Institut du Cerveau et de la Moelle épinière, Hôpital de la Pitié-Salpêtrière, INSERM UMRS 975—CNRS UMR 7225-UPMCParis, France; ^11^Department of Psychology, Faculty of Humanities and Social Sciences, University of ZagrebZagreb, Croatia; ^12^Physiology of Cognitive Processes, Max Planck Institute for Biological CyberneticsTübingen, Germany

**Keywords:** self-organized criticality, human intracranial recordings, spike train analysis, highly parallel recordings, spiking neural networks, multiunit activity, cortex, monkeys

## Abstract

In self-organized critical (SOC) systems avalanche size distributions follow power-laws. Power-laws have also been observed for neural activity, and so it has been proposed that SOC underlies brain organization as well. Surprisingly, for *spiking* activity *in vivo*, evidence for SOC is still lacking. Therefore, we analyzed highly parallel spike recordings from awake rats and monkeys, anesthetized cats, and also local field potentials from humans. We compared these to spiking activity from two established critical models: the Bak-Tang-Wiesenfeld model, and a stochastic branching model. We found fundamental differences between the neural and the model activity. These differences could be overcome for both models through a combination of three modifications: (1) subsampling, (2) increasing the input to the model (this way eliminating the separation of time scales, which is fundamental to SOC and its avalanche definition), and (3) making the model slightly sub-critical. The match between the neural activity and the modified models held not only for the classical avalanche size distributions and estimated branching parameters, but also for two novel measures (mean avalanche size, and frequency of single spikes), and for the dependence of all these measures on the temporal bin size. Our results suggest that neural activity *in vivo* shows a mélange of avalanches, and not temporally separated ones, and that their global activity propagation can be approximated by the principle that one spike on average triggers a little less than one spike in the next step. This implies that neural activity does not reflect a SOC state but a slightly sub-critical regime without a separation of time scales. Potential advantages of this regime may be faster information processing, and a safety margin from super-criticality, which has been linked to epilepsy.

## Introduction

Avalanches, earthquakes, and forest fires are all cascades of activity in otherwise quiescent systems (Gutenberg and Richter, 1944; Bak et al., 1987; Drossel and Schwabl, 1992; Frette et al., 1996; Dickman et al., 2000). Most of the time, the size of these cascades, or avalanches, is small, but sometimes avalanches are large enough to span the entire system. The size *s* of an avalanche is the number of units activated during a cascade, and interestingly, the distribution *f(s)* of avalanche sizes in the systems mentioned above precisely follows a power law:

(1) $$f\text{​}\left(s\right)\sim s^{-\tau }$$

where τ is the critical exponent. Critical exponents determine the macroscopic behavior of a system, and indicate the system’s universality class (Wilson, 1975).

Power law distributions are characteristic for second-order phase transitions, where the system is in a “critical” state. If the system evolves to reach a critical state without fine-tuning of control parameters, the system is termed *self-organized critical* (SOC) (Bak et al., 1987; Jensen, 1998; Nagler et al., 1999; Beggs and Plenz, 2003; Frigg, 2003; Beggs and Timme, 2012; Pruessner, 2012).

SOC models show avalanches or cascades of activity across their units, which may arise from simple local interactions (Bak et al., 1987; Drossel and Schwabl, 1992; Olami et al., 1992). These avalanches can include all units in the system. However, most avalanches are small or intermediate in size. Note that avalanches of size one, i.e., only one unit is active and no further activity is triggered, have the highest chance of occurring (see Equation 1). Overall, avalanches are not characterized by an average size, i.e., the size distribution is scale-free, and only the true size of the system restricts the avalanche size range.

In nervous systems, scale-free properties have been observed in local field potentials (LFP), electro- and magnetoencephalographic (EEG, MEG) activity, and BOLD signals (Linkenkaer-Hansen et al., 2001; Beggs and Plenz, 2003; Petermann et al., 2009; Hahn et al., 2010; Ribeiro et al., 2010; Tetzlaff et al., 2010; Friedman et al., 2012; Poil et al., 2012; Tagliazucchi et al., 2012; Priesemann et al., 2013; Shriki et al., 2013). They have been found in different preparations, ranging from cultures to *in vivo* preparations, and across different species and phyla: leeches, rats, cats, monkeys, and humans (Linkenkaer-Hansen et al., 2001; Beggs and Plenz, 2003; Mazzoni et al., 2007; Pasquale et al., 2008; Petermann et al., 2009; Priesemann et al., 2009, 2013; Hahn et al., 2010; Ribeiro et al., 2010; Tetzlaff et al., 2010; Friedman et al., 2012; Poil et al., 2012; Tagliazucchi et al., 2012; Shriki et al., 2013). The prevailing hypothesis is that scale-free neural activity arises from SOC behavior (Linkenkaer-Hansen et al., 2001; Beggs and Plenz, 2003; Mazzoni et al., 2007; Beggs, 2008; Pasquale et al., 2008; Petermann et al., 2009; Shew et al., 2009; Hahn et al., 2010; Ribeiro et al., 2010; Tetzlaff et al., 2010; Friedman et al., 2012; Poil et al., 2012; Tagliazucchi et al., 2012; Gal and Marom, 2013; Shriki et al., 2013). However, there are also studies that reported deviations from scale-free activity: Neural activity was shown to exhibit sub-critical and super-critical behavior during development *in vitro* (Pasquale et al., 2008; Tetzlaff et al., 2010; Friedman et al., 2012); and there are also studies in which *in vivo* neural activity appeared as sub-critical (Bedard et al., 2006; Priesemann et al., 2013). Thus, healthy brains seem to be capable of organizing themselves into a range of states that are not necessarily SOC.

Nevertheless, because neural activity from coarse scale measures (e.g., population spikes, LFP, MEG, BOLD) often do show power law scaling, the same was expected for more basic constituents of neural activity, namely the spiking activity. Surprisingly, however, spike avalanches often deviated from power law scaling (Bedard et al., 2006; Pasquale et al., 2008; Hahn et al., 2010; Tetzlaff et al., 2010). In fact, to the best of our knowledge, there is not a single study that demonstrated power laws for spikes in awake animals. The deviations from power law scaling in previous studies were attributed either to sub- or super-critical states (Pasquale et al., 2008; Tetzlaff et al., 2010), or to subsampling effects (Ribeiro et al., 2010). Subsampling refers to the technical constraint that only a fraction of all neurons in a given area can be measured. Subsampling can impede the observation of power law distributions in SOC models (Priesemann et al., 2009, 2013; Ribeiro et al., 2010; Girardi-Schappo et al., 2013) and hence a critical system can be misinterpreted as sub- or super-critical (Priesemann et al., 2009). Therefore, subsampling effects need to be taken into account when interpreting spike avalanches.

An important property of SOC systems, which is potentially absent in neural activity, is the separation of time scales (STS) (Bak et al., 1987; Drossel and Schwabl, 1992; Clar et al., 1996; Dickman et al., 2000; Pruessner, 2012; Hartley et al., 2013) whereby pauses between avalanches last much longer than the avalanches proper. For example, forest fires last for a much shorter time than it takes to regrow the forest. Similarly, earthquakes are much more rapid than the time it takes to build shear stress through plate tectonics (Drossel and Schwabl, 1992; Clar et al., 1996, 1999; Baiesi and Paczuski, 2004). Likewise, in the classical sandpile model, scale-free avalanche distributions are observed only if the grains are dropped at a low enough rate (Vespignani and Zapperi, 1997, 1998). This low rate of external input, called drive, is a necessary condition for the long pauses and hence for SOC (Bak et al., 1987; Drossel and Schwabl, 1992; Clar et al., 1996; Dickman et al., 2000; Pruessner, 2012; Hartley et al., 2013).

**Figure 1:**
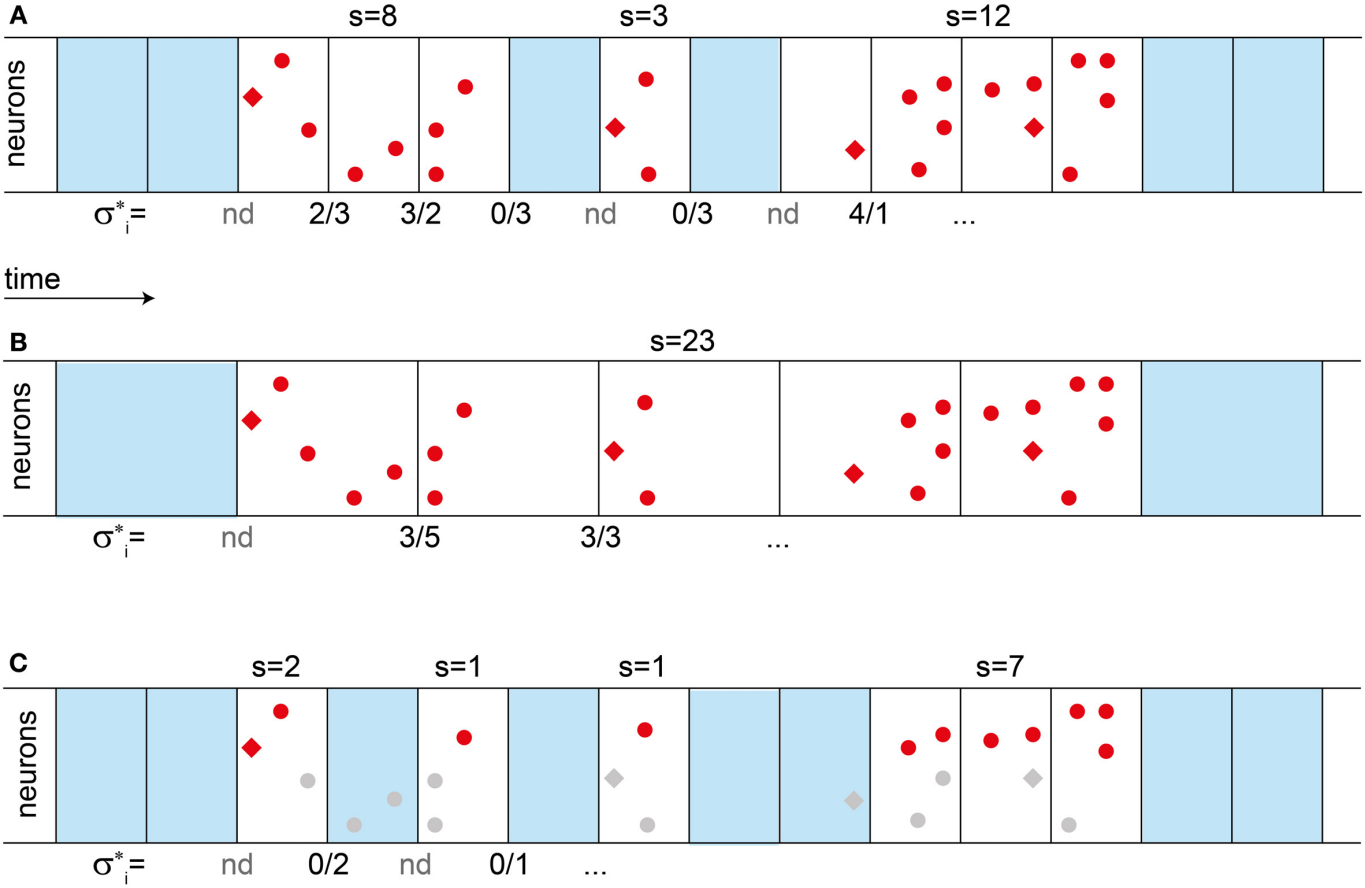
Definition of avalanches sizes, branching parameter σ^*^, and their change with bin size. (A) To define avalanches, temporal binning (boxes) is applied to a sequence of spikes (red dots and diamonds). Empty bins are marked in blue. An avalanche is an ensemble of spikes in a sequence of non-empty bins. Its size *s* is the total number of spikes, as indicated above the bins. The branching parameter σ^*^_i_ is the ratio between the number of spikes in one bin, divided by the number of spikes in the previous bin, as indicated below the bins. If the previous bin was empty, σ_i_ is “not defined” (nd). The estimated branching parameter σ^*^ for an experiment is the average over all σ^*^_i_, σ^*^ = <σ_i_>. **(B)** When increasing the bin size, the observed avalanches can become larger, since pauses “disappear”. The branching parameter σ^*^ also changes with the bin size. **(C)** Under subsampling, only a fraction of the units are recorded (red dots), while others are missed (gray). Thereby subsampling can split a single avalanche into several parts. **(A–C)** In the model, spikes are either triggered externally by some drive (red diamonds), or they are evoked by presynaptic activity (red dots). If a second avalanche is triggered while the first one is still active [last avalanche in **(A)**], then the two avalanches cannot be told apart and are evaluated as if they were a single one.

Neither the neural activity we analyzed here, nor that from previous studies of neural avalanches showed STS: There were no long pauses in the neural activity which could be seen as natural separations between avalanches. Without such pauses, unambiguous detection of the beginning and the end of an individual avalanche is not possible. Hence, the method of temporal binning had been introduced as a workaround (Beggs and Plenz, 2003) (Figure 1). Here, the choice of the bin size determines what is considered to be a pause between avalanches. Consequently, avalanche sizes necessarily change with the choice of the bin size (see e.g., Beggs and Plenz, 2003; Priesemann et al., 2009, 2013; Hahn et al., 2010). This implies that also the avalanche size distributions and, more importantly, power law exponents change with the choice of bin size (Beggs and Plenz, 2003; Priesemann et al., 2013). This is in marked contrast to fully sampled SOC systems, in which the power law exponents do not change under temporal binning as a result of STS. These differences have to be considered when comparing neural activity *in vivo* to that of classical SOC models.

As indicated above, in classical SOC systems each avalanche is separated from the next one by a long pause. In contrast, in *driven* SOC systems, i.e., SOC systems without STS, avalanches can meet, merge, intermingle, and split up: They form a mélange. As we demonstrate in this paper, neural activity indeed resembles such a mélange of avalanches instead of well-separated ones.

To investigate the differences between *in vivo* and model activity, we analyzed spike avalanches recorded in awake rats and monkeys, anesthetized cats, and LFP avalanches recorded in humans, and compared these *in vivo* avalanches to avalanches from an established SOC model (Bak-Tang-Wiesenfeld model) (Bak et al., 1987; Dunkelmann and Radons, 1994; Priesemann et al., 2009, 2013), and to those from a stochastic branching model (Harris, 1963; Haldeman and Beggs, 2005).

## Results

As a widely held belief states that mammalian nervous systems operate in a SOC state, we first briefly recapitulate the theoretically expected avalanche statistics in this state by example of a SOC model and a critical stochastic branching model. We then show that all of the analyzed neural avalanches *in vivo* showed clear deviations from the expected statistics.

The remainder of the results then demonstrates how two simple and neurophysiologically well-motivated conceptual changes in the models can serve to align model and *in vivo* activity with respect to a large set of measured quantities.

### Differences between neural dynamics *in vivo* and SOC

**Figure 2:**
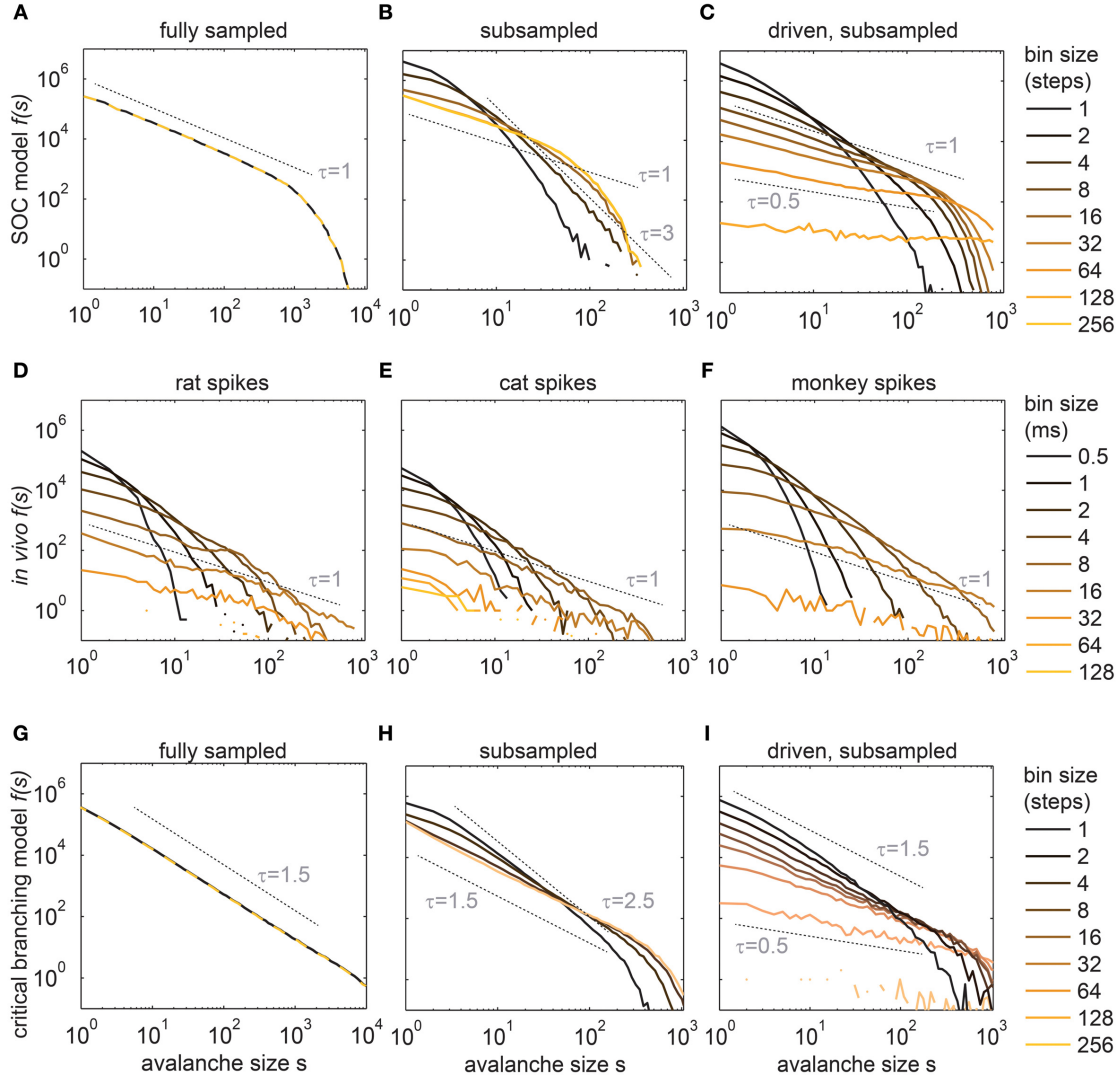
**Avalanche size distributions *f(s)* changed with the bin size for the *in vivo* spike trains (D–F), and for the subsampled models (B,C,H,I)**. **(A)** *f(s)* of the SOC model under full sampling did not depend on the bin size. **(B)** Under subsampling (*N* = 100 neurons), *f(s)* of the same SOC model changed with small bin sizes only. **(C)** In the driven model (*h* > 0) *f(s)* changed for all bin sizes. *h* was chosen such that the population rate *R* of the 100 sampled model neurons matched *R* of the experiments (*R* ≈ 320 Hz). **(D–F)** *f(s)* recorded in the hippocampus (awake rat), the visual cortex (anesthetized cat), and the prefrontal cortex (awake monkey). **(G–I)** shows the same as **(A–C)**, but for a critical branching model instead of the SOC model. Dashed lines indicate potential power law slopes to guide the eye. All *f(s)* are logarithmically binned and *f(s)* is in absolute counts.

The first example model is a simple neural network model, which is known to have SOC properties (Bak et al., 1987). Furthermore, this SOC model has been shown to match LFP avalanches in monkeys and humans (Priesemann et al., 2009, 2013). In our study, the model consisted of 2500 non-leaky integrate-and-fire neurons arranged as a 50 by 50 grid with nearest neighbor connections of synaptic strength α = 1 (see Methods). In this model, spikes are either evoked by activity from presynaptic neurons, or by a random external input to a neuron. This input is termed drive and has a rate *h*. For *h* → 0 and α = 1, this model obeys local energy conservation (Bonachela et al., 2010), and is equivalent to the well-known SOC Bak-Tang-Wiesenfeld model (Bak et al., 1987). *h* → 0 is necessary for a model to be SOC (Vespignani and Zapperi, 1997, 1998; Dickman et al., 2000), because it guarantees the obligatory STS. *h* → 0 is implemented by applying external input only when there is otherwise no activity in the model. The input triggers an avalanche, i.e., a cascade of events. The size *s* of an avalanche is defined as the total number of spikes evoked by a single input spike. This model is known to show a power law for *f(s)* with slope τ ≈ 1 (Figure 2A), and a cutoff at *s* ≈ 1000 (Bak et al., 1987). This cutoff reflects the finite size of the model (Bak et al., 1988; Kadanoff et al., 1989; Ktitarev et al., 2000).

To later demonstrate that our conclusions are not specific to the SOC model above, we simulated a second model, namely a stochastic branching model (see Methods) (Harris, 1963; Haldeman and Beggs, 2005). Like the SOC model, it was simulated with 2500 neurons, but in contrast to the SOC model, the *k* = 4 postsynaptic neurons were chosen randomly at each step. Activity propagated stochastically, i.e., an active neuron activated each of its *k* postsynaptic neurons with probability *p* = α/k. Like the SOC model, this model is critical for α = 1, and sub- (super-) critical for α < 1 (α > 1). The critical stochastic branching model with STS also showed a power law distribution for *f(s)*, but with a different critical exponent (τ = 1.5, Figure 2G).

The results for the stochastic branching model and the SOC model were qualitatively the same for all measures used below. The similarity also held when the models were modified analogously. Therefore, in the following, we mainly report results for the SOC model.

Our critical models were contrasted with highly parallel recordings from awake rats (hippocampus), awake monkeys (prefrontal cortex), and from an anesthetized cat (visual cortex area 18). The avalanche distributions *f(s)* from these *in vivo* spike recordings were all very similar, but clearly differed from those obtained from the fully sampled critical models (compare Figures 2D–F with A,G). In particular, the *in vivo f(s)* neither followed a power law, in contrast to what is expected for a SOC system, nor an exponential distribution, as would be expected for independent Poissonian activity (Figures S1 and S2 show the *in vivo f(s)* for each experiment in double-logarithmic and log-linear scales, respectively). Quantitatively, the *f(s)* were best fit in 16 out of 17 experiments by a lognormal distribution

$$f(s)\sim e^{-\frac{{\left(ln(s)-\mu \right)}^{2}}{2\sigma^{2}}}$$

with parameters μ = 0.89 ± 0.25 and variance σ^2^ = 1.2 ± 0.1, given a bin size of 1 average inter event interval (<IEI>) (see Clauset et al., 2007; Priesemann et al., 2013 for details). Based on these parameters the maximum of *f(s)* was at *s* = 0.87 ± 0.38 (mean ± SD), which means that *f(s)* was monotonically decreasing. Two alternative distributions, namely stretched exponentials and power laws with cutoff, also provided reasonable fits, with likelihoods ~1% worse than the one for the lognormal distribution.

Interestingly, all *in vivo* avalanche distributions were similar despite changes in the population rate *R* by a factor of 50 (from 37 to 1560 Hz) across the 17 experiments (Figures S1, S2).

Note that some of the *f(s)* of the rat experiments could also be approximated by a power law, but at most for one selected bin size (Figure S3A). When slightly changing the bin size, the *f(s)* clearly deviated from power law scaling (Figure S3B). This is in stark contrast to the behavior expected for SOC systems.

A second striking difference between critical models and *in vivo* activity was that the *in vivo f(s)* changed with the bin size across a range from 0.5 to 128 ms. The reason for the bin size dependence was that *in vivo* recordings showed pauses of variable length between the spikes, while SOC activity showed only the long pauses between avalanches, which are due to STS. In order to introduce pauses of variable length into the model avalanches, one can apply subsampling and drop STS (see next two sections).

### Subsampling introduces pauses at short time scales

Subsampling refers to the problem that we are far from being able to sample all spikes from all neurons, even for a single brain area (Figure 1C). Thus, for a careful comparison between *in vivo* recordings and models, the activity from the models should be subsampled in the same manner as in the experiments. Because in each experiment around 100 neurons were recorded in parallel, in the model we constrained the sampling also to *N* = 100 randomly chosen neurons out of the 2500. We fixed the subsampling by the number of neurons, and not the fraction, because running these critical models with millions of neurons is beyond our computational capacities, and because the qualitative results did not change in larger models, i.e., when decreasing the fraction (see below).

When applying subsampling, the model avalanche size distribution *f(s)* changed with bin size (Figures 2B,H). A change in bin size affected *f(s)*, because subsampling introduces apparent pauses in a single avalanche (Figure 1C). These apparent pauses were relatively short compared to the duration of an avalanche, and compared to the pauses between avalanches on the full model (by definition of STS). Therefore, when subsampling, *f(s)* changed only with small bin sizes but stopped to change its shape with larger ones (Figures 2B,H).

These results also held when using a larger model and sampling the same number of neurons, i.e., a smaller fraction of neurons. In this case, the distance and hence the traveling time of avalanches between sampled neurons became larger and longer, and the inter spike intervals became unrealistically long. Nonetheless, at large bin size, a similar fraction of small avalanches was observed (due to STS). As a consequence, *f(s)* also stopped changing like in smaller models, and never became as flat as the *in vivo f(s)*. Hence, the behavior of a larger model was the same as that of smaller ones, but on a longer time scale.

Subsampling the SOC model did not only introduce a dependence of *f(s)* on the bin size, it also affected the cutoff of *f(s)*. Thereby, the absolute value of the cutoff became more similar for the model and the *in vivo f(s)* (Figures 2B,H).

In sum, acknowledging subsampling effects in the model allowed for a better match between the model and the *in vivo* activity, but only for small bin sizes up to a few milliseconds. For larger bin sizes, the *in vivo f(s)* continued to become flatter, while the model *f(s)* stopped to change their shape. This indicated that a modification to the model dynamics itself was necessary to match *in vivo* activity.

### An increase in drive rate *h* creates a mélange of avalanches

**Figure 3:**
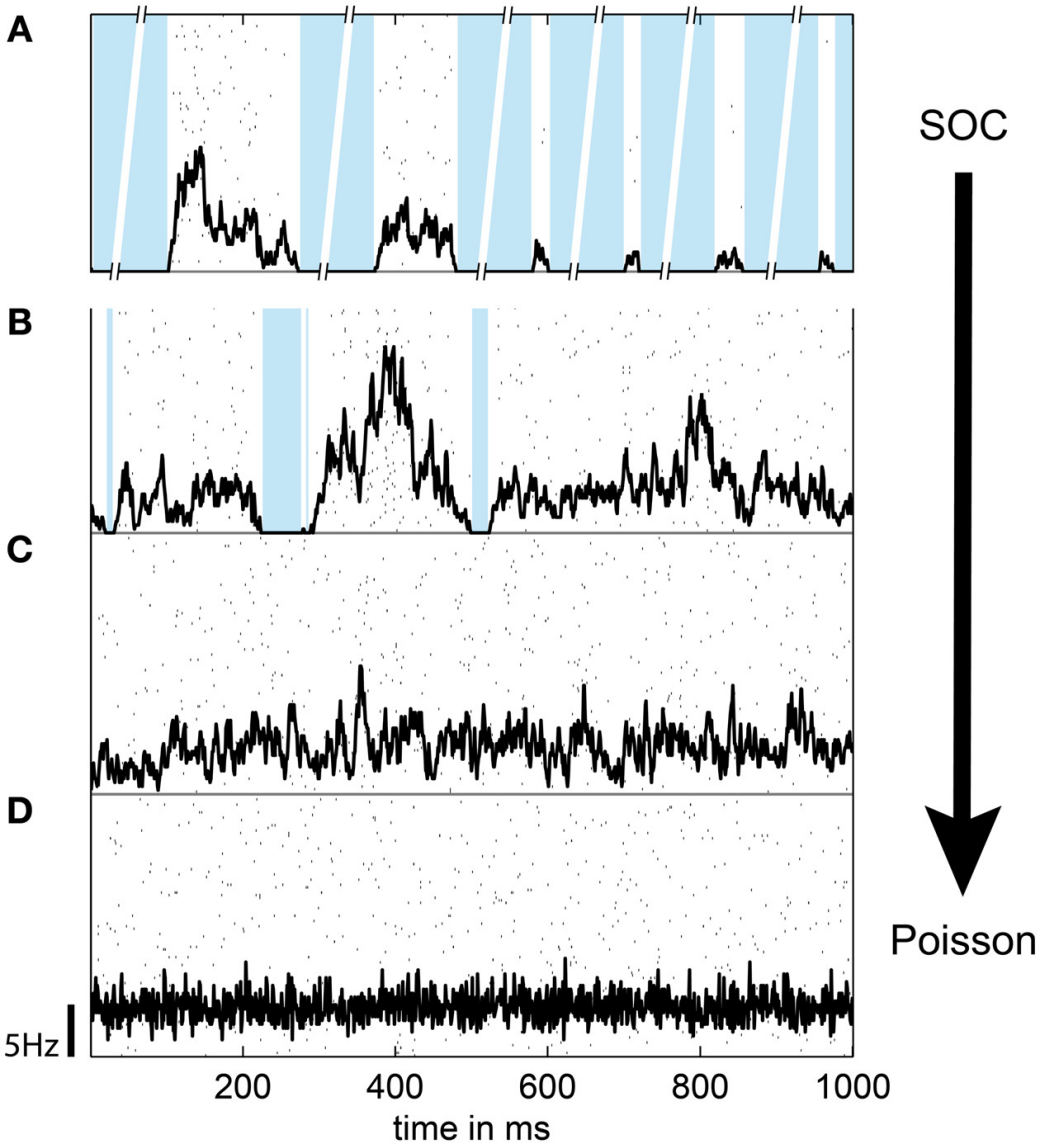
**The population spike rate of the (modified) SOC model depended on the connection strength α and the rate of input spikes *h* (drive)**. *h* and α were balanced such that the rate of each unit was *r* = 5 Hz, except for **(A)**, where α = 1 and *h* → 0 (SOC model). In **(A)**, the broken axes indicate that the pauses between subsequent avalanches are much longer than the avalanche proper (separation of time scales). **(B)** α = 1, *h* = 0.02 Hz, *r* = 5 Hz (driven SOC). **(C)** α = 0.95, *h* = 0.5 Hz, *r* = 5 Hz (driven sub-critical). **(D)** α = 0, *h* = *r* = 5 Hz (Poisson). In **(A–D)**, the population rate time course is indicated in black; the scale bar indicates the firing rate per neuron. Black dots show the spike raster from 100 randomly chosen units; the blue background denotes pauses, i.e., none of the 2500 neurons spiked. Note the absence of pauses in **(C,D)**.

We hypothesized that *in vivo* and SOC activity differed because SOC models have STS (Vespignani and Zapperi, 1997, 1998; Dickman et al., 2000), which is necessarily absent *in vivo*. STS can be eliminated from the models by increasing the drive rate *h*. We increased *h* in such a way that the model population rate *R* matched the *in vivo* population rate under subsampling (*h* = 0.02 Hz, and *R* = 320 Hz; single neuron rate *r* in the model: *r* = *R/N* = 3.2 Hz). In this *driven* SOC model, the avalanches were no longer separated by long pauses (Figure 3B). Instead, at any point in time, avalanches could start, meet, intermingle, split into branches, or die out (Figures 1, 3B). In such a mélange of avalanches, single avalanches can no longer be tracked.

The mélange of avalanches in the driven model hardly showed any pauses when all neurons were sampled (Figure 3B). However, under subsampling, pauses were more frequent. Thus, subsampling allowed for an extraction of apparent avalanches by applying temporal binning (Figure 1). Note that these apparent avalanches do not correspond to the avalanches observed in classical SOC models in which avalanches are separated by long pauses, and are thereby defined unambiguously. However, the apparent avalanches from the driven models are conceptually the same as those extracted from *in vivo* recordings because avalanches in both cases are extracted with the same method.

As expected for the *driven*, subsampled SOC model, *f(s)* changed with all bin sizes (Figures 2C,I), and thereby resembled the *in vivo f(s)* much better than the original SOC model (Figure 2).

### Driven critical and driven sub-critical states

In the following, we address the question whether subsampling and the elimination of STS is sufficient to match the model activity with the *in vivo* activity, or whether it is necessary to introduce in addition deviations from criticality.

**Figure 4:**
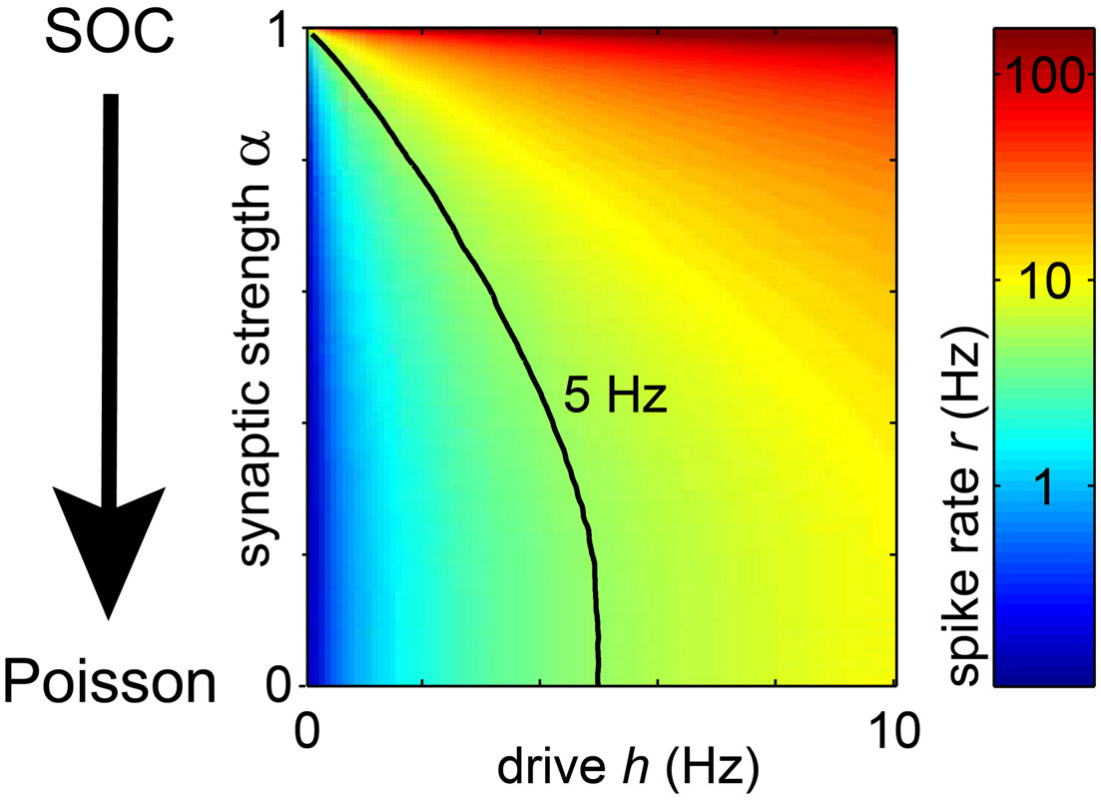
**In the model, the spike rate *r* of a unit depended on the synaptic strength α and the rate of input spikes (*h*)**. With increasing *h* or α, the rate of each unit increased. The black line indicates the parameter combination of α and *h*, for which *r* = 5 Hz.

To tune models away from criticality, we made use of the fact that SOC and branching models are only critical in the conservative limit (α = 1) (Harris, 1963; Bonachela and Muñoz, 2009; Bonachela et al., 2010). Hence, by introducing dissipation (α < 1) these models can be made sub-critical. In fact, the model dynamics showed a smooth transition from the “driven SOC” state (α = 1) to pure Poisson activity (α = 0) (Figures 3, 4) with decreasing α. In principle, a decrease in α also decreased the firing rate *r* of each unit. To still maintain a constant firing rate *r*, a concomitant increase in the drive rate *h* was applied. In this way, the model could make the transition from driven SOC to Poissonian activity without a change in *r* (Figure 4, black line). Given a fixed *r*, a decrease in α decreased the variability of the models population rate (Figure 3).

To understand which network dynamics between driven critical and Poissonian accounted best for the *in vivo* spike avalanches, we identified those measures in the model which depended most sensitively on α *under subsampling:* α had a prominent effect on the avalanche size distribution *f(s)*, in particular how *f(s)* depended on the bin size. We quantified this below using the following avalanche measures: the mean avalanche size (<*s*>), the frequency of avalanches of size *s* = 1 (*f*(*s* = 1)), and the estimated branching parameter σ^*^. The way in which these measures changed with the bin size depended sensitively on α. In addition, we estimated the scaling exponent β of the “detrended fluctuation analysis” (DFA) (Peng et al., 1994, 1995; Kantelhardt et al., 2002). (Note that the scaling exponent (β) is often denoted as α in the literature). The results of these analyses are presented in detail below, and compared one by one to the *in vivo* results.

### The mean avalanches size

**Figure 5:**
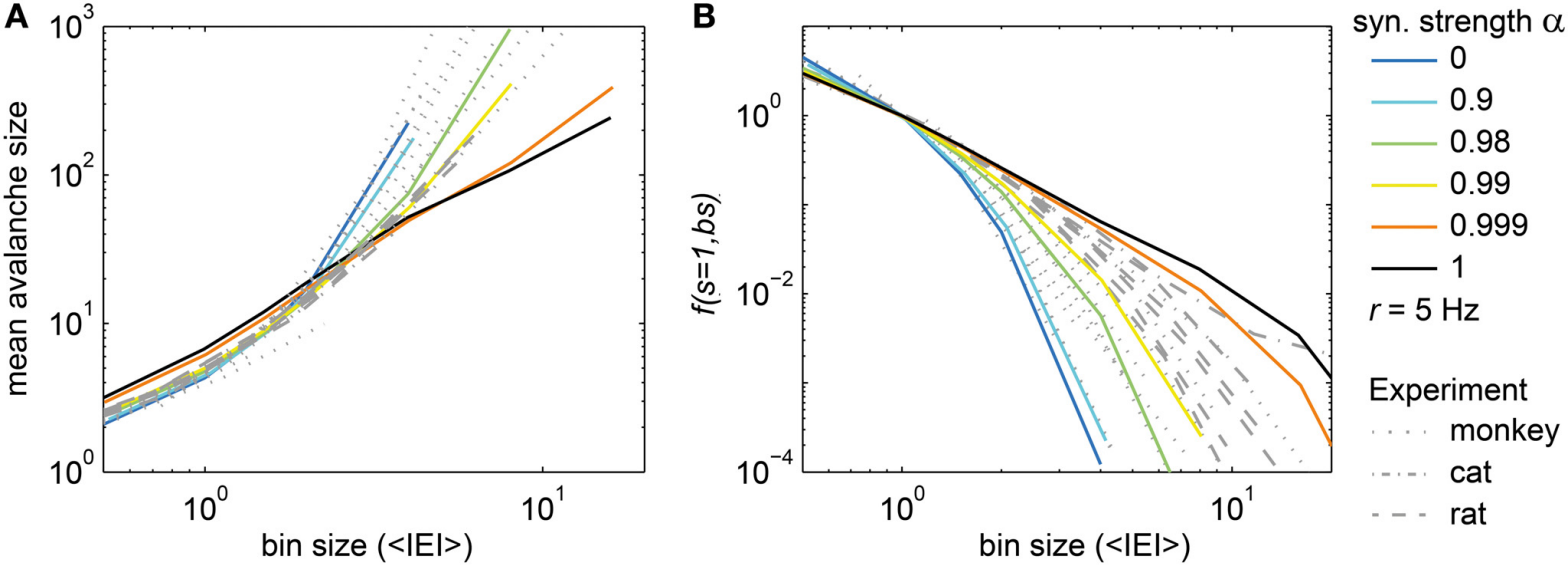
**Two new avalanche measures. (A)** The mean avalanche size and **(B)** the frequency of avalanches with size *s* = 1, *f*(*s* = *1*, bs), changed with the bin size (*bs*) in the model (colored) and in the experiments (gray). The colored lines show *f*(*s* = *1, bs*) for the model with varying synaptic strength α. In the model, the drive rate *h* was adjusted such that each neuron spiked with *r* ≈ 5 Hz. In **(B)**, *f*(*s* = *1, bs*) was normalized such that *f*(*s* = *1, bs* = *1* <*IEI*>) = 1.

The mean avalanche size (<*s*>) from the subsampled model followed a power law with increasing bin size for α = 1 (driven SOC), and followed an exponential for α = 0 (Poissonian activity) (Figure 5A). For intermediate values of α, the relation changed gradually.

For the experiments, the observed <*s*> at a given bin size depended strongly on the population spike rate *R* that varied considerably between experiments (*R* ranged from 37 Hz to 1.5 kHz). To diminish the impact of *R*, we used a normalized bin size, i.e., a bin size in units of average inter-event-intervals (1 <*IEI*> = 1/*R*). Using the normalized bin size, the <*s*> of all experiments overlapped (Figure 5A, gray lines). However, the <*s*> did not follow a power law with changing bin size *in vivo*, in contrast to the driven critical model. In fact, the *in vivo* <*s*> was best matched by the <*s*> of the driven, sub-critical models (α ≈ 0.99). Thus, comparing the *in vivo* and model <*s*> indicated that spike avalanches resembled a driven sub-critical regime more closely than a driven SOC state.

### The frequency of avalanches of size one

The frequency of avalanches of size *s* = 1, *f*(*s* = 1, *bs*) quantifies how *f(s)* decayed with the bin size (*bs*) at *s* = 1, i.e., how the intercept of *f(s)* with the y-axis in Figure 2 changed. *f(s)* at *s* = 1 was equally spaced from bin size 1 to 32 ms for the driven critical models under subsampling (Figures 2C,I) which is remarkable as it corresponds to a power law behavior of *f*(*s* = 1, *bs*) for the driven SOC model (black line in Figure 5B; note that the x-axis here is in <*IEI*>, and 1 <IEI> = 2 ms in the model). For the sub-critical models (α < 1), *f*(*s* = 1, *bs*) decayed more steeply than a power law. For the Poissonian case (α = 0), it followed an exponential. In this respect, *f*(*s* = 1, *bs*) and <*s*> showed similar behaviors with α.

**Figure 6:**
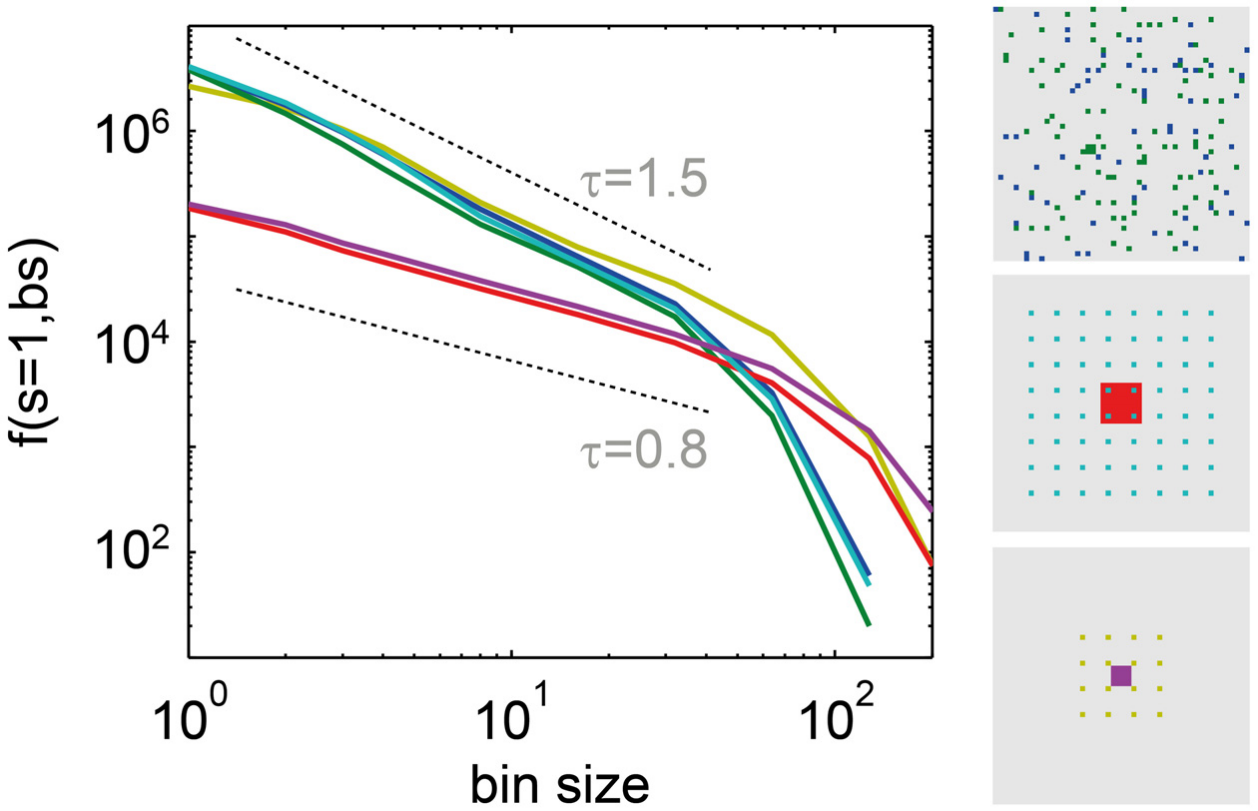
**The frequency of single events *f*(*s* = 1, *bs*)**. Decreased with the bin size (*bs*) as a power law, independently of the subsampling set in the driven SOC model (α = 1, *r* = 5 Hz). The subsampling set is indicated in the right part of the figure. It was chosen as follows: blue *f*(*s* = 1, *bs*): sampling 64 random units; green *f*(*s* = 1, *bs*): sampling 100 random units (both, blue, and green units together); red and turquoise: sampling 8 × 8 units arranged in a grid with distance 1, and distance 5, respectively; pink and beige: sampling 4 × 4 units with distance 1, and 5, respectively.

*f*(*s* = 1, *bs*) is a promising new measure to assess criticality under subsampling, because in contrast to many other measures, its behavior did not change with the subsampling strategy: For the driven SOC model, it showed power law scaling independently of the number and spatial arrangement of the sampled units (Figure 6). However, the slope of the power law did change due to the model’s next-neighbor topology: With smaller distances between sampled sites, the power laws became flatter (red and pink traces in Figure 6). For the stochastic branching model, the same results held, but the power law slopes did not change under subsampling, owing to the model’s random topology.

The *in vivo f*(*s* = 1, *bs*) did not follow a power law (Figure 5B, gray lines), and for most cases did not follow an exponential dependency either (Figure 5B). The best approximation for the *in vivo f*(*s* = 1, *bs*) was the driven, slightly sub-critical model (α ≈ 0.99). This is in agreement with the results for <*s*>.

The precise value of α necessary to achieve the best match between model and experiments potentially depended on a number of factors (e.g., finite size effects). However, the main result that <*s*> and *f*(*s* = 1, *bs*) observed *in vivo* followed neither a power law nor an exponential distribution excludes both, critical and Poissonian states of operation.

### The branching parameter σ

A widely used measure to estimate whether the *in vivo* avalanches reflected a driven SOC brain state is the branching parameter σ^*^, which has been used in many past studies about neural avalanches to test whether the brain was SOC (Beggs and Plenz, 2003; Beggs, 2007; Plenz and Thiagarajan, 2007; Priesemann et al., 2009, 2013; Shew et al., 2009; Klaus et al., 2011; Shriki et al., 2013). The analysis of σ^*^ was initially inspired by the theory of branching processes (Harris, 1963), in which σ = 1 guarantees that a branching process is critical. Note, however, that *estimating* σ^*^ from data may yield misleading results, because σ^*^ depends on various factors such as the bin size (Beggs and Plenz, 2003; Priesemann et al., 2013), the subsampling geometry (Priesemann et al., 2009), and STS (i.e., *h* → 0 vs. *h* > 0). We next show how σ^*^ depended on these factors in our models, and then use these results to estimate whether the *in vivo* avalanches might reflect a SOC state.

**Figure 7:**
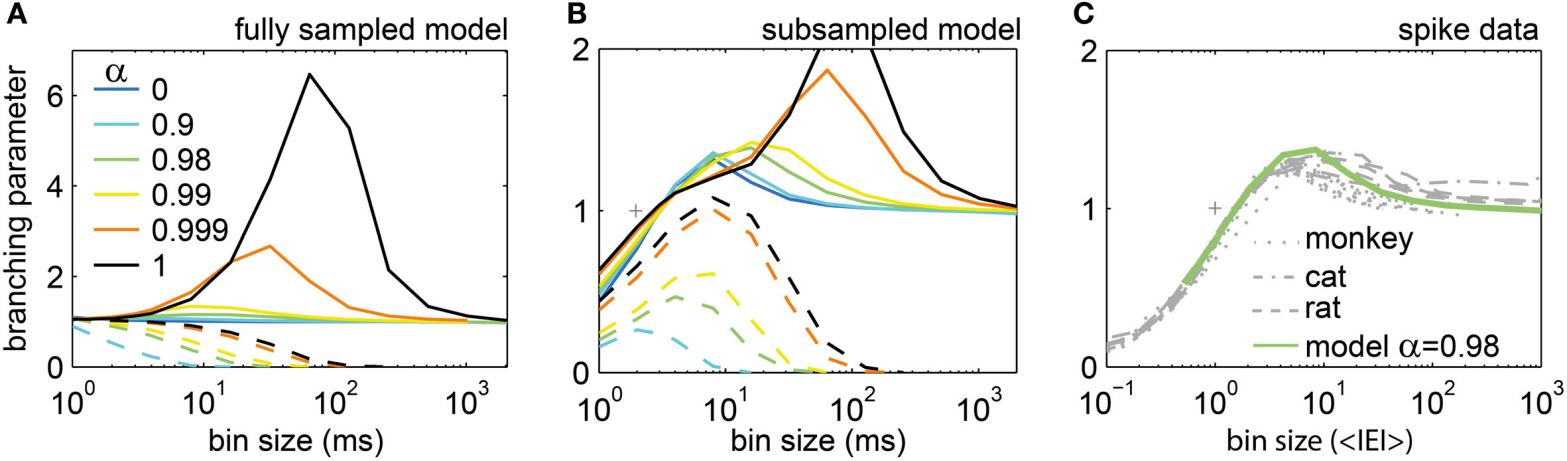
The estimated branching parameter σ^*^ changed with bin size. (A,B) In the model, σ^*^ depended on the synaptic strength α and the bin size. For the driven model, the spike rate was fixed to *r* = 5 Hz (full lines), while for the model with separation of time scales the drive was infinitesimal small (*h* → 0; dashed lines). For *h* → 0 and α = 1, the model is SOC (black dashed lines). **(A)** Results for the fully sampled model. **(B)** Results for subsampling *N* = 100 neurons from the model. **(C)** σ^*^ for the spiking activity recorded in monkeys, cats, and rats varied with the bin size, but was very similar across species and experiments. It was well approximated by the driven model with α = 0.98 (green line).

For the modified SOC model, we expect that σ equals α. For the second model we used, i.e. the stochastic branching model, we *know* by definition of the model that σ equals α. However, when estimating σ^*^ in this model by applying temporal binning to the model activity, finding the expected σ^*^ = α was the exception, not the rule (Figure S4; results were very similar to the ones for the SOC model in Figure 7). In addition, σ^*^ changed with the bin size, although the model parameter σ proper is obviously bin size independent (Figures 7, S4). Although the estimated σ^*^ failed to approximate the true σ, σ^*^ may still be a viable approach to compare model and *in vivo* activity in the following. Since the results for both models were basically the same, we again focus on the results for the modified SOC model.

With STS, σ^*^ always approached zero for large bin sizes independently of model state and subsampling approach (dashed lines in Figures 7A,B, S4). For intermediate bin sizes and under subsampling, σ^*^ varied widely. σ^*^ tended to be smaller for smaller α, but the absolute value of σ^*^ apparently cannot serve as an indicator for the state of the system (Figures 7A,B). Thus, under most analysis conditions, the estimated σ^*^ did not show the intended result (σ^*^ = α). Note that in theory, σ^*^ should not change at all with the bin size.

Without STS (full lines in Figures 7A,B, S4), σ^*^ was ≤1 for small bin sizes, ≥1 for intermediate bin sizes, and approximated unity for large bin sizes – independently of the state of the model. This shows that the widely held assumption that an estimated σ^*^ > 1 (σ^*^ < 1) corresponds to a super-critical (sub-critical) state of the system is likely incorrect, especially for the ubiquitous scenario of subsampling.

Although the expected σ^*^ = 1 is neither unique to critical systems, nor indicative of criticality, σ^*^ and its dependence on the bin size still reflect the intrinsic dynamics of the system. Therefore, comparing σ^*^ between *in vivo* and model activity may still help to indicate the state of the system. Note that to estimate the *in vivo* σ^*^ we used the normalized bin size (in <*IEI*>) to account for the different population rates *R* in the experiments. σ^*^ was very similar across all experiments (Figure 7C) despite a 50-fold difference in *R*. This indicates once again that neural avalanches *in vivo* hardly differ across mammalian species (from rats to monkeys), across brain structures (from hippocampus to prefrontal cortex), and across cognitive states (from anesthetized to awake behaving animals).

Given the complex dependence of σ^*^ on the bin size, how can σ^*^ be used to estimate the precise state of the neural network? First, for all *in vivo* avalanches, σ^*^ approximated unity for large bin size (Figure 7C). However, this simply indicates that spiking activity *in vivo* lacks STS. Second, the maximum of σ^*^ under subsampling may be an indicator of the state. The maximum of σ^*^ increased with increasing α. For α = 1, σ^*^ showed a maximum of ≈3 at *bs* ≈100 ms. [The same values held for the stochastic branching model (Figure S4)]. For the experiments, the maximum value of σ^*^ was only around 1.4. Overall, the best match for the *in vivo* σ^*^ was achieved by the driven, slightly sub-critical models (α ≈ 0.98). This result is in line with the previous results for *f*(*s* = 1, *bs*) and <*s*>.

### The scaling exponent β

**Figure 8:**
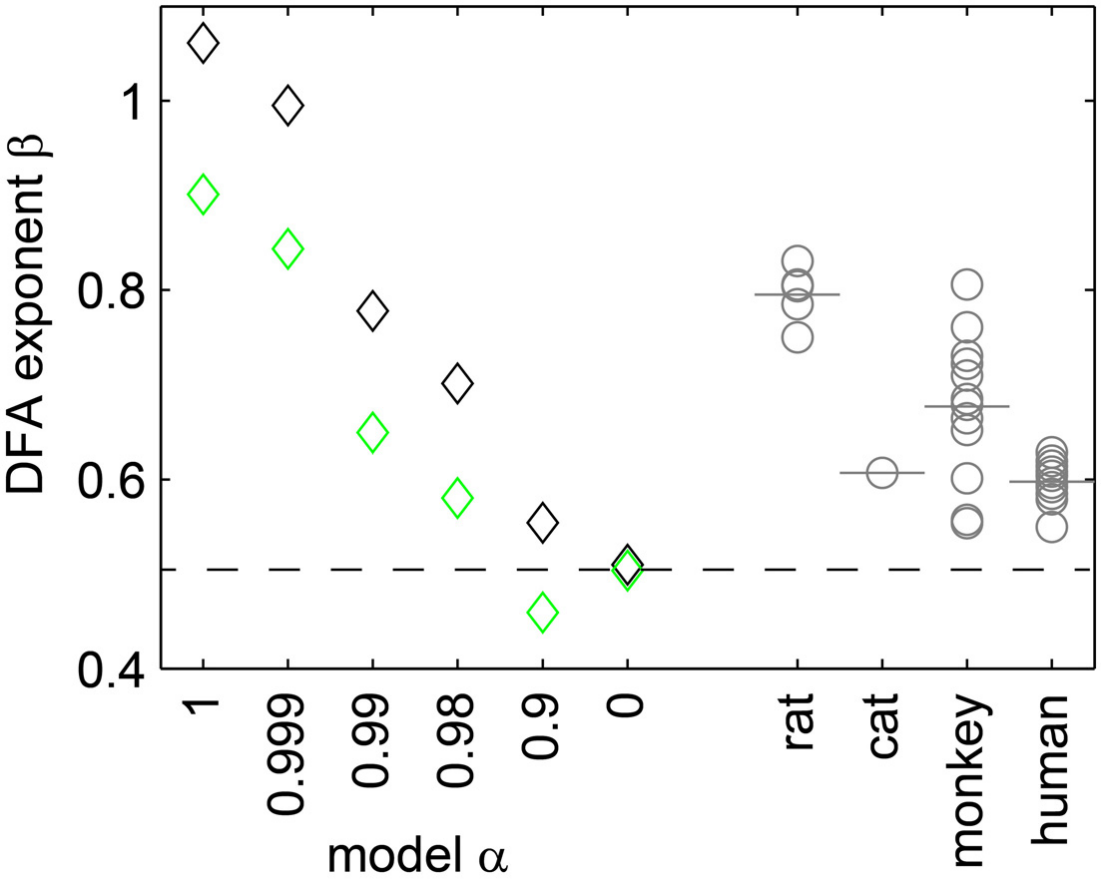
**The exponent β of the DFA**. Depended on the synaptic strength α in the model (diamonds), and was affected by subsampling (black: fully sampled model; green: subsampled model). For the experiments, β (gray circles) and the respective mean values (gray bars) ranged between 0.55 and 0.9.

In DFA, the scaling exponent β quantifies the memory decay in a time series. β = 0.5 indicates that a time series has no memory (uncorrelated); β ≈ 1 indicates 1/f (pink) noise; and β ≈ 1.5 Brownian noise. We estimated β for the population rate time series of the model (*r* = 5 Hz), and for each experiment. As expected, under full sampling the model with α = 1 showed β ≈ 1 (Figure 8, black diamonds); with decreasing α, β decreased as well; and for α = 0 (Poisson), we found β ≈ 0.5. Qualitatively, the same results held under subsampling, but β tended to be underestimated (Figure 8, green diamonds).

The *in vivo* activity showed neither β = 1 nor β = 0.5, but β ranged between 0.55 and 0.9. These β values correspond to those of the sub-critical, driven model with 0.98 ≤ α < 0.999.

**Figure 9:**
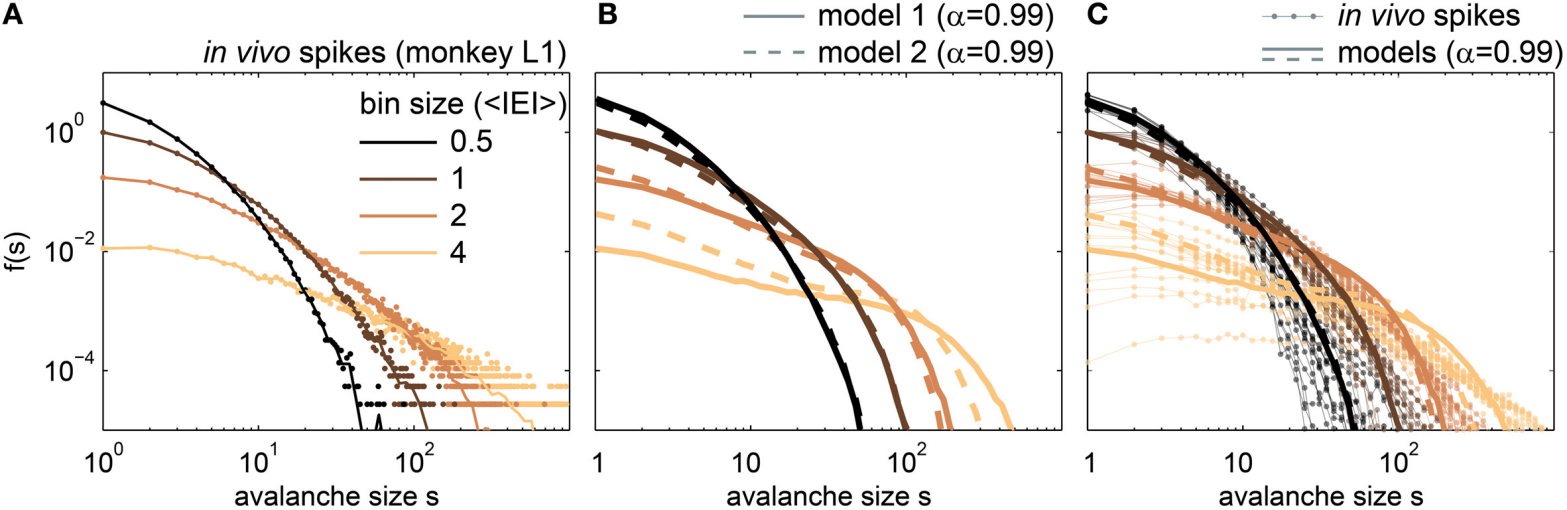
**Avalanche size distributions *f(s)* for *in vivo* spikes and for spikes from the driven, sub-critical models**. **(A)** *f(s)* of one awake monkey. Dots indicate the raw *f(s)*, while lines are the *f(s)* with logarithmic binning. **(B)** *f(s)* for the driven, sub-critical models with α = 0.99, and *r* = 5 Hz; model 1 denotes the modified SOC model (full lines), and model 2 the stochastic branching model (dashed lines). **(C)** *f(s)* of all in *in vivo* spike recordings (rat, cat, monkey), together with the *f(s)* of the driven, subcritical models (same as in **B**). All bin sizes were in average inter event intervals (<IEI>), and *f(s)* were normalized such that *f*(*s* = *1, bs* = *1*): = 1.

All the above measures indicated that driven, slightly sub-critical models provided the best match to *in vivo* spike avalanches. Most of these measures were derived from the avalanche size distribution, and hence we expect a good match between the *in vivo f(s)*, and the *f(s)* of the driven models with α ≈ 0.99. Indeed, given a normalized bin size, both sub-critical models fitted the *in vivo f(s)* well (Figure 9). The small differences for large *s* (*s* > 100) may potentially be overcome by applying a more realistic drive instead of uncorrelated Poissonian drive, for example one that reflects the statistics of neural activity (as lined out here), or the statistics of our environment (Field, 1987; Van der Schaaf and van Hateren, 1996; Simoncelli and Olshausen, 2001; Sinz et al., 2009).

### LFP avalanches in humans

Approximate power law distributions have been reported for coarse measures of neural activity, such as population spikes, LFP, EEG, MEG, and BOLD activity (Linkenkaer-Hansen et al., 2001; Beggs and Plenz, 2003; Petermann et al., 2009; Hahn et al., 2010; Ribeiro et al., 2010; Tetzlaff et al., 2010; Friedman et al., 2012; Poil et al., 2012; Tagliazucchi et al., 2012; Priesemann et al., 2013; Shriki et al., 2013). In the following, we show that also LFP recordings in humans indicate a driven, slightly subcritical regime, despite their approximate power law scaling of *f(s)*.

**Figure 10:**
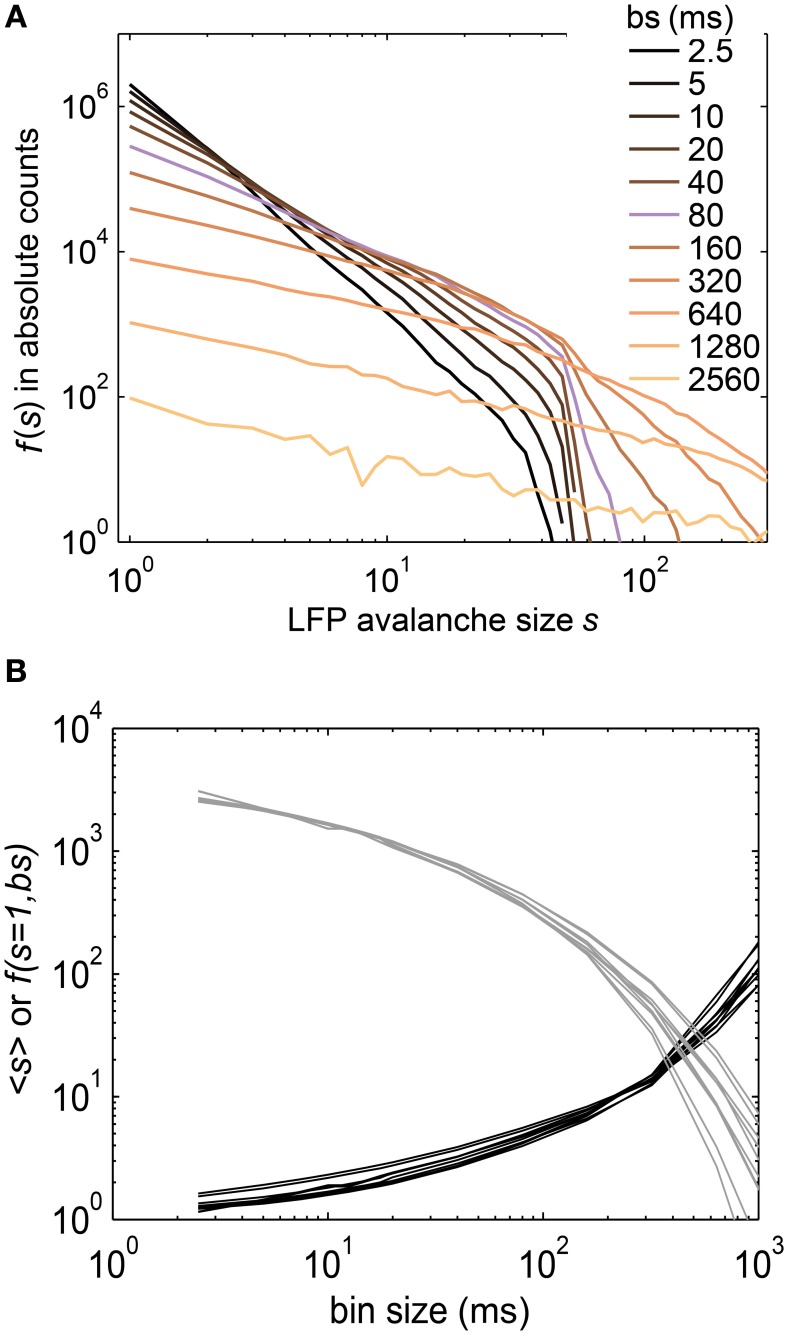
**(A)** The size distribution *f(s)* of LFP avalanches from intracranial depth electrodes in humans followed power laws. The slope of the power laws changed with the bin size (see legend). The bin size was changed over a 1000-fold range, from sampling resolution (400 Hz, i.e., 2.5 ms) to “gluing” everything together at *bs* ≈ 2500 ms. The bin size closest to one inter event interval is marked in purple (*bs* = 80 ms, see Methods). **(B)** Neither the mean avalanche size (<*s*>), nor the frequency of avalanches of size *s* = 1, *f(s = 1, bs)*, showed a power law. Each line represents the results for one recording session (<*s*> in black, *f*(*s* = *1, bs*) in gray).

LFPs were recorded using intracranial depth electrodes from five human subjects. Each subject had between 44 and 63 recording contacts implanted. From these recordings, we extracted avalanches of enhanced activity (see Methods and Priesemann et al., 2013). The LFP *f(s)* closely followed a power law (Figure 10A), and the slope of the power law decreased with increasing bin sizes. This is in contrast to SOC systems in which the slope does not change with temporal binning (Figures 2A,G), and indicates that LFP avalanches, like the spike avalanches, lack clear STS.

**Figure 11:**
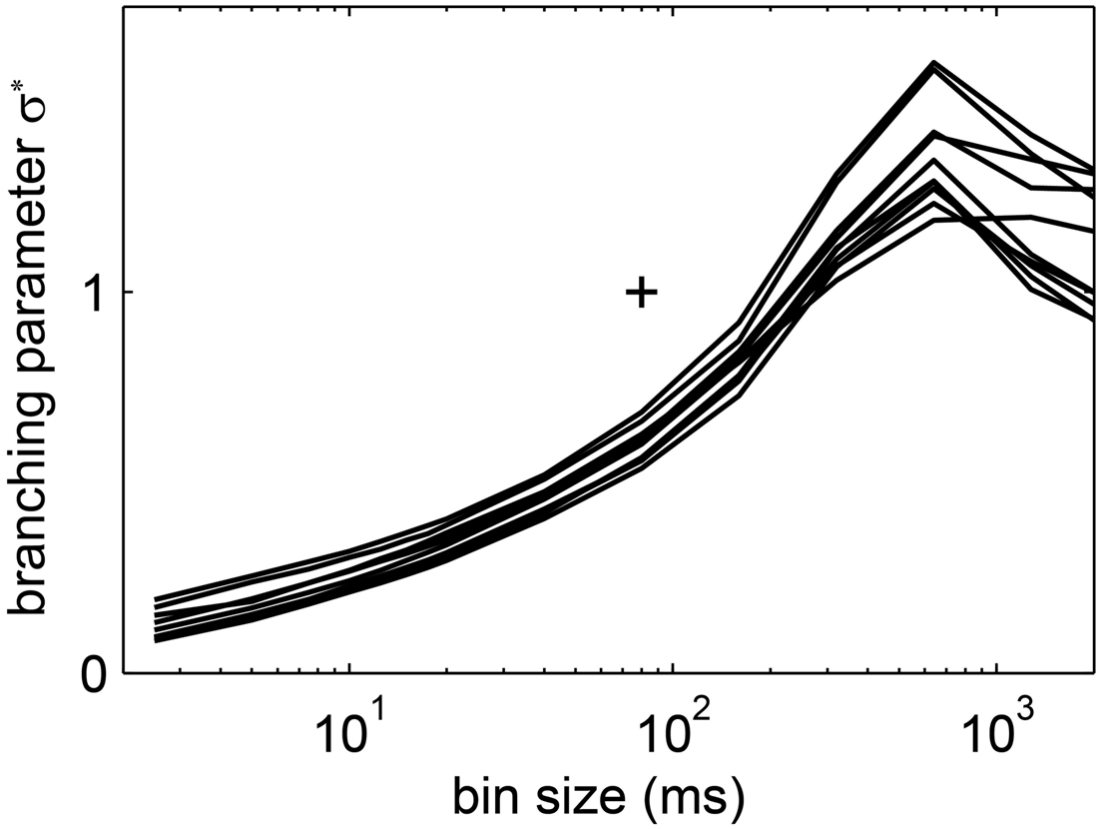
The estimated branching parameter σ^*^ from the LFP avalanches in humans changed with the bin size. Each of the lines shows the results for one recording session. (+) indicates σ^*^ = 1 and *bs* = 80 ms ≈ 1 <IEI> to guide the eye.

In general, the LFP *f(s)* showed a better approximation to power law scaling than any of the spike avalanche distributions (Figures 2, 10). Despite an approximate power law scaling for *f(s)*, all the other measures we used here [i.e., <*s*>, *f*(*s* = 1, *bs*), σ^*^, and β] indicated a sub-critical regime: The <*s*> and the *f*(*s* = 1, *bs*) both deviated from power law scaling (Figure 10B); the branching parameter did not show a pronounced peak (Figure 11); and the scaling exponent β of the DFA was smaller than unity (mean(β) = 0.6; Figure 7). This is in line with our previous study on the same data (Priesemann et al., 2013), and with our results for spiking activity. In sum, despite approximate power-law scaling in *f(s)*, all the other measures indicated a driven, slightly sub-critical regime on the level of LFP activity.

## Discussion

This study challenges the hypothesis that mammalian brains operate in a SOC state, as has been repeatedly suggested (Linkenkaer-Hansen et al., 2001; Beggs and Plenz, 2003; Haldeman and Beggs, 2005; Levina et al., 2007a; Hsu et al., 2008; Pasquale et al., 2008; Stewart and Plenz, 2008; Petermann et al., 2009; Priesemann et al., 2009; Shew et al., 2009; Hahn et al., 2010; Ribeiro et al., 2010; Tetzlaff et al., 2010; Poil et al., 2012; Tagliazucchi et al., 2012; Shriki et al., 2013). Despite these claims, evidence for SOC was found lacking for spiking data, which are generally considered an important and reliable marker of neural activity. To test the SOC hypothesis, we therefore analyzed *in vivo* spiking activity from three mammalian species and local field potential recordings from the human brain using established measures of criticality, and also novel ones that are robust to common shortcomings of experimental data, such as subsampling. We particularly focused on systematic changes of these measures with the choice of the bin size.

Spike avalanches from rats, cats, and monkeys, and LFP avalanches from humans showed deviations from the behavior expected for SOC, thereby contradicting the SOC hypothesis. To reproduce the *in vivo* results and provide potential explanations for their deviations from SOC, we modified the models capable of critical behavior. We found a close match between *in vivo* and model behavior (1) if those models were subsampled, and (2) if the STS – a fundamental property of SOC systems – was eliminated, and (3) if the models were tuned to a sub-critical regime. As these results generalized over two very different models, we interpret results from the *in vivo* recordings here as evidence that mammalian nervous systems operate in a driven, sub-critical regime. This regime, albeit not critical, was, however, remarkably similar across species and experimental conditions.

### Universal behavior of spike avalanche distributions across recording areas, vigilance states and species

The observed avalanche size distributions *f(s)* were similar across species and recording areas (hippocampus in rats, visual cortex in cats, prefrontal cortex in monkeys). A similar universality of *f(s)* across recording areas has been reported by Ribeiro and colleagues (hippocampus, somatosensory cortex, and visual cortex in rats) (Ribeiro et al., 2010). Thus, avalanche activity seems to be independent of the function and the precise anatomy of an area. This might either indicate that avalanches are not a sensitive measure of neural dynamics, or that activity propagation must follow principles that are independent of the specific role that a brain area plays in information processing. The first argument is not likely applicable, since avalanches change under data shuffling and they sensitively reflect the correlation structure in the data (e.g., Figure 1 in Priesemann et al., 2013). The second argument might indeed hold. Hence, the challenge is to identify the principle that gives rise to these apparently universal spike avalanche distributions. This principle may in fact be very simple. As discussed below, our modified SOC model, as well as a simple branching model, suggests that on average one spike gives rise to a little less than one subsequent spike, and that quiescence in the population activity is prevented by “input spikes” which trigger avalanches at a low rate. This principle differs from SOC, where one spike *on average* gives rise to exactly one subsequent spike, and the rate of input spikes approaches zero (STS). As a consequence, SOC activity shows only one avalanche at a time, while the driven, slightly sub-critical regime shows instead a mélange of avalanches.

### Empirical avalanche distributions rule out the critical and the poisson states

Let us first summarize the conclusions that can be drawn from the analyses of the *in vivo* spike avalanches alone, without referring to modeling. For *f(s)*, neither was the power law scaling found, that is characteristic for SOC, nor did the novel measures (*f*(*s* = 1, *bs*), <s>) support the hypothesis of critical behavior. Thus, the hypothesis that spike avalanches show signs of SOC can be ruled out. In addition, we can rule out the hypothesis of largely independent Poissonian behavior of the spiking units (that is often used in models), because in this case the avalanche distributions should have shown exponential behavior, which was not observed. We therefore conclude that spiking activity is neither (self-organized) critical nor Poissonian.

### Limitations of the models and measures

The SOC model used here was admittedly simple – it comprised neither inhibitory connections nor leakage in the neurons; synaptic connections had a homogeneous nearest-neighbor topology and were all of identical strength α. We chose this model because the basic variant (σ = 1, *h* → 0; i.e., the Bak-Tang-Wiesenfeld model; Bak et al., 1987) is extensively studied in the context of SOC (De Menech et al., 1998; Jensen, 1998; Vespignani et al., 1998; Dickman et al., 2000; Dhar, 2006; Pruessner, 2012). The second model we used was a stochastic branching model (Harris, 1963; Haldeman and Beggs, 2005). It was set up to be comparable to the SOC model, but had a random topology, and the activity propagated stochastically with *p* = α/*k*. In this model, the number of connections *k* hardly affected the results (see also Haldeman and Beggs, 2005).

For both models, the avalanche dynamics was qualitatively similar. Hence, the model results were not specific to the topology (local vs. random), the number of connections *k*, and the precise spike propagation mechanisms (deterministic vs. stochastic). In contrast, implementing leaky model neurons may hinder SOC altogether (Bonachela and Muñoz, 2009; Bonachela et al., 2010). This in itself is an argument against the hypothesis that neural activity is SOC, but it could still be “quasi-critical” (Bonachela and Muñoz, 2009; Bonachela et al., 2010). However, our results indicate sub-criticality.

We note that the power law scaling observed for the novel measures (*f*(*s* = 1, *bs*), <s>) in the critical models has not been derived analytically yet. However, in both critical models the novel measures showed power law scaling despite the different topology and the different spike propagation rules, and hence we expect this behavior to be characteristic for critical dynamics. Still, for now these measures can only be used as tools to compare model and *in vivo* dynamics, and not for determining scaling laws.

### On the plausibility of external drive

Spike and LFP avalanches recorded in rats, cats, and primates were best matched by a *driven* sub-critical model. The drive in the model consisted of input spikes, i.e., of spikes not caused by presynaptic spikes from within the model. Given their importance for a successful match between *in vivo* and model activity, we may ask what the *in vivo* counterpart of the input spikes in the models could be. *In vivo*, such input spikes can be provided by at least three sources—by sensory input elicited by stimuli in the outside world, from brain structures other than the one under consideration, or by internal activation which presumably occurs spontaneously. Such spontaneous activity can for example be generated by pacemaker cell activity (Selverston, 2008; Longtin, 2013), or vesicle fusion at a presynaptic terminal without a preceding spike (Fredj and Burrone, 2009). With all these known input sources *in vivo*, it came as no surprise that the model required input spikes (i.e., drive) to be able to match *in vivo* activity.

### Input spikes most likely do not constitute a large fraction of the observed activity

The fraction of “input spikes” (drive) among all the spikes of the model is negligible at criticality (α = 1). This fraction, given a constant spike rate *r*, increases with tuning toward sub-criticality (α < 1), until *all* spikes are input spikes in the Poisson state (α = 0), and none arises from synaptic transmission. For example, in the driven, slightly sub-critical model (α = 0.99), only one in ~3600 spikes was an input spike. To illustrate this number, imagine a neuron that spikes with a rate of 1 Hz. This neuron fires spontaneously (i.e., an “input spike”) only once an hour. This example is simplistic, because it assumes that the input is homogeneous, however, it illustrates well that the fraction of input spikes (from the external world, other brain structures, or of stochastic origin; see above) in the driven, sub-critical regime that reproduced the *in vivo* findings is extremely small compared to the overall level of activity.

### Conceptual considerations on the analysis of neural avalanches and the critical state

While we have so far discussed how *in vivo* spike avalanches suggest a driven sub-critical regime of operation for mammalian nervous systems, several neglected but important conceptual issues with the analysis of neural avalanches surfaced in this study. These are discussed in the following.

### The term “avalanche” refers to different entities in SOC models and in the analysis of neural data

Although it is rarely fully acknowledged, the term “avalanche” refers to different entities for activity in SOC models and for neural activity. In SOC models, an avalanche is a cascade of events that originates from a *single* input event (Bak et al., 1987). Subsequent avalanches are always separated by pauses (STS). In contrast, for neural activity, avalanches are defined using temporal binning (Beggs and Plenz, 2003), because neural activity lacks clear pauses that could naturally serve to define the beginning and end of an avalanche. Such avalanches can be defined on any spike time series, irrespective of its origin. Consequently, “binning-dependent avalanches” do not correspond to classical SOC avalanches. Although these two types of “avalanches” are different entities, it is customary to use the same term when referring to any one of them. In the present study, we analyzed the “binning-dependent” avalanches in both cases, in the driven models and in the *in vivo* activity. This justifies a comparison between model and *in vivo* activity, and was also necessary as binning-dependent avalanches are the de-facto standard in the analysis of neural systems, although previous studies frequently alluded to SOC avalanches.

### Avalanche definitions in highly parallel recordings

For neural activity, avalanches are commonly defined using temporal binning, and this definition relies on pauses. We can expect that physiologically relevant pauses (i.e., pauses of a few ms) vanish in spike recordings, when activity of a large number of neurons is recorded in parallel. For example, if each neuron spikes with 1 Hz, sampling only 100 neurons in parallel would frequently produce pauses that are several milliseconds long. However, when sampling thousands or even millions of such neurons, pauses would probably be absent. Without pauses, neither the classical nor the binning-dependent avalanche definition is applicable, and consequently, alternative approaches to assess criticality have to be established. Currently, these approaches threshold the activity and thereby introduce pauses (e.g., Spasojević et al., 1996; Papanikolaou et al., 2011; Poil et al., 2012). As an alternative approach, we propose to apply systematic subsampling. Both approaches allow using the binning-dependent avalanche definitions again.

### Can we determine a specific critical exponent for neural data?

Avalanche size distributions of critical branching processes have an exponent of *τ* = 1.5 (Harris, 1963). Since branching processes have some resemblance with propagation of neural activity, it was hypothesized that neural avalanches should also show τ = 1.5. Indeed, τ = 1.5 has been observed (Beggs and Plenz, 2003, 2004; Stewart and Plenz, 2008; Hahn et al., 2010; Priesemann et al., 2013), but only for specific bin sizes. For example, Beggs and Plenz showed in their seminal work that τ = 1.5 holds for one specific bin size (4 ms), but when changing the bin size from 1 to 16 ms, the exponent decreased from 2 to 1.2 (Beggs and Plenz, 2003). Similarly, for the LFP avalanches shown here, τ = 1.5 was observed only for a bin size of ~80 ms, and with varying the bin size from 2.5 ms to ~2.5 s, the exponent changed from 3 to 1 (Figure 10A) (Priesemann et al., 2013). Changes in τ were also observed in the driven, subsampled SOC model (Figure 2C). Thus, drive and subsampling may underlie the variation of τ in experiments as well. However, irrespective of its origin, it is an open question how to reconcile the variation of τ in neural data with the fixed τ in critical systems. One proposal is to use a specific bin size for neural data, namely one average inter-event-interval (<IEI>) (Beggs and Plenz, 2003). However, there is no theoretical underpinning yet why this bin size should be preferred over others, and even for using this bin size, τ was found to be 1.8 in spike avalanches in anesthetized cats (Hahn et al., 2010), instead of 1.5. Thus, in neural data, there is not a unique τ, and therefore there is no specific critical exponent for neural activity, which would allow to link neural activity to a universality class.

Since neural avalanche distributions change with the bin size (Beggs and Plenz, 2003; Priesemann et al., 2009, 2013; Benayoun et al., 2010; Hahn et al., 2010), we side with Benayoun et al., who “do not read any significance into the particular slope observed. [… ] In our view, any good model of neural avalanches must reproduce the variability in the observed slope of the power law with temporal bin width.” (Benayoun et al., 2010) Though we here did not observe power laws for the *in vivo f(s)*, our model could reproduce the *in vivo* spike *f(s)* and their change with temporal binning. It could also reproduce the bin-size dependent changes of novel and established measures of avalanche dynamics (*f*(*s* = 1, *bs*), <s>, σ^*^, DFA exponent). To the best of our knowledge, this is the first model that matched not only the avalanche properties for a single bin size, but also their changes with changing bin size.

### Subsampling effects in the assessment of criticality

Subsampling is unavoidable in spike avalanche recordings *in vivo*, and is helpful when comparing neural activity to model activity (Priesemann et al., 2009). However, subsampling was also shown to complicate criticality analysis because it can distort avalanche measures (Priesemann et al., 2009, 2013; Ribeiro et al., 2010). To overcome this problem, we here developed avalanche measures that are not distorted by subsampling. One example is the bin size dependence of the frequency of avalanches of size one (*f*(*s* = 1, *bs*)). This measure robustly showed power-law scaling in the driven SOC states, and exponential scaling in the Poisson state, independent of subsampling strategies (Figure 6). Therefore, we propose to use *f*(*s* = 1, *bs*) as a robust measure for criticality analysis.

Subsampling effects can appear very strong if one uses a fixed bin size, e.g., 1 ms as in Ribeiro et al. (2014). We used instead a normalized bin size, which accounts for the problem that the population rate *R* changes with the number of sampled neurons. Using a normalized bin size diminished subsampling effects, and also allowed for a comparison to the *in vivo* recordings.

### Finite size effects in criticality assessment

The finite size of the critical models limited the correlation lengths in space and time and thereby caused the cutoff in *f(s)* (Figures 2A,G). In analogy, the finite size is expected to also have caused – in the driven critical models – the cutoff at large bin size in the novel measures (*f*(*s* = 1, *bs*), <s>). Since finite size effects decrease with increasing system size, and since the *in vivo* spikes were recorded in a far larger system than our model spikes, finite size effects are unlikely to account for the deviations from power law scaling found for the *in vivo* activity.

In critical models, the finite size can change the value of α, for which the model is critical. For example, Eurich et al. (2002) showed for their model that the critical α depended on the model size *L* as α_crit_ = 1 − *L*^−0.5^. Thus, their finite size models with α → 1 were super-critical and showed peaks in their *f(s)*. This was not the case for our critical models. Our models, in contrast, appeared to be slightly sub-critical at α = 1. This is probably due to the open boundary conditions we used in contrast to Eurich et al. Hence, since the finite size made our models at most sub-critical but not super-critical, there is no concern that the observed match of model and *in vivo* results at values of α < 1 is due to finite size effects.

### Different types of critical phase transitions exist

To better understand criticality and potential deviations from it, it is also important to define which type of criticality one refers to. Critical phase transitions can occur for example for the transitions from order to chaos (Bertschinger and Natschläger, 2004; Haldeman and Beggs, 2005; Boedecker et al., 2012; Lizier, 2013), from non-oscillatory to oscillatory regimes (Linkenkaer-Hansen et al., 2001; Poil et al., 2012), from replay to non-replay of spatio-temporal patterns (Scarpetta and de Candia, 2013), and from a regime with finite to one with potentially infinite avalanche sizes (Bak et al., 1987; Drossel and Schwabl, 1992; Olami et al., 1992; Eurich et al., 2002; Beggs and Plenz, 2003; Haldeman and Beggs, 2005; Levina et al., 2007a,b, 2009), as known from branching processes (Harris, 1963). One study has found that the transitions to chaos and to potentially infinite avalanches coincide in their model (Haldeman and Beggs, 2005), but it is unclear whether this finding generalizes to other systems. We here want to emphasize that our model showed a transition to potentially infinitely large avalanches.

### Consequences for information processing and stability of brain dynamics

After having discussed evidence from *in vivo* spike avalanche distributions for a driven, sub-critical mode of operation, and after having clarified conceptual issues, we now turn to the question of what consequences these findings may have on information processing and dynamic stability in the mammalian brain.

### Sub-criticality, super-criticality, and stability

Criticality is characterized by a power-law distribution of its avalanche sizes. This indicates that avalanches of any size can occur; even close to infinite-size avalanches may occur, provided that the system is large enough to sustain them. Infinite-size avalanches do occur in the super-critical regime, and have been linked to epileptic seizures (Hsu et al., 2008; Meisel et al., 2012). Such infinite avalanches produce runaway activity, and could thereby impair normal brain activity. Therefore, it is unlikely that it would be good for a normally functioning brain to be super-critical. Sub-criticality, in contrast, never shows infinitely large avalanches, and thus offers a safer regime for brain operation. Thus, a *slightly* sub-critical regime allows the brain to avoid runaway activity, while still allowing moderate activity propagation, and maintaining most of the possible computational advantages that come with criticality (Haldeman and Beggs, 2005; Kinouchi and Copelli, 2006; Beggs, 2008; Shew et al., 2009; Shew and Plenz, 2013).

### Drive and information processing

There may be good reason why neural activity *in vivo* does not show a STS for its avalanches: When eliminating the STS, avalanches run in parallel, meet, and intermingle. Thereby, the *rate* of computations may be increased compared to the SOC state. In addition, the presence of multiple, potentially interacting avalanches, may enable collision-based computation, which is one fundamental way of information modification (Lizier, 2013). Thus, a driven state may increase the rate and capacity of neural information processing *in vivo*.

## Conclusions

Our analysis of *in vivo* data indicated that the mammalian brain is not SOC because *in vivo* spiking activity differed fundamentally from activity expected for SOC. Instead, the mammalian brain apparently self-organizes to a slightly sub-critical regime without an STS. Mechanistically, such a driven, sub-critical regime shows a mélange of avalanches, while SOC systems, in contrast, are characterized by temporally separated avalanches. Operating in a slightly sub-critical regime may prevent the brain from tipping over to super-criticality, which has been linked to epilepsy. Regarding computational capabilities, which have been reported to be optimal for SOC, a slightly sub-critical regime only deviates little from SOC and therefore its computational capabilities may still be close to optimal, while the non-zero drive in general may allow for a higher rate of information processing. Taken together, a driven, slightly sub-critical regime may strike a balance between optimal information processing and the need to avoid runaway activity.

## Methods

### Self-organized critical model

The SOC neural network model we used here is the Bak-Tang-Wiesenfeld model (Bak et al., 1987), and modified versions of it. Translated to a neuroscience context, the model consisted of 2500 non-leaky integrate and fire neurons. A neuron *i* spiked if its membrane voltage *V*_*i*_(*t*) reached a threshold Θ:

$$If\;V_{i}\text{​}\left(t\right)>\Theta ,\;V_{i}(t+1)=V_{i}(t)-4$$

Θ was set to Θ = 0 for convenience. Note that the choice of Θ does not change the activity of the model at all. The model neurons were arranged on a 2D lattice, and each neuron was connected locally to its four next neighbors, i.e., the coupling strength α_*ij*_ = α for all four next neighbors of neuron *i*, and α_*ij*_ = 0 else.

$$V_{i}(t+1)=V_{i}(t)+\sum_{j}\alpha_{ij}\cdot \delta (t-T_{j})+H(t)$$

The time *t* was updated in ms (i.e., 1 ms effective synaptic delay). *T*_*j*_ denoted the spike times of neuron *j*, and *H(t)* was a function which set a neuron above threshold with a certain Poisson rate *h*. *h* represented the “drive” in the context of SOC. Note that the neurons at the edges and corners of the grid had only 3 and 2 neighbors, respectively. This model is equivalent to the well-known Bak-Tang-Wiesenfeld model (Bak et al., 1987) if *h* → 0 and α = 1. In contrast, for α = 0, the model represented independent Poisson units which spiked with rate *r* = *h*.

Subsampling (Priesemann et al., 2009) was applied to the model by sampling the activity of 100 randomly selected neurons only, and neglecting the activity of all other neurons. To simulate specific subsampling effects, the sampled neurons were not chosen randomly, but arranged in specific configurations (see Figure 6, right part). Here the sampled neurons were arranged to have very small or very large distances. For the small distances, 4 × 4 or 8 × 8 neurons from a compact, central subset were sampled (Figure 6, red and pink), and for the large distances, 4 × 4 or 8 × 8 neurons with distance 5 grid units between them were sampled (Figure 6, turquoise and beige).

### Stochastic branching model

In addition to the SOC model, we also simulated a classical stochastic branching model. In this model, a branching process (Harris, 1963; Haldeman and Beggs, 2005) was mapped on a grid of neurons. An active neuron activated each of its *k* postsynaptic neurons with probability *p* = α · 1/*k*. As in the SOC model, this model was critical for α = 1 in the infinite size limit, and subcritical (supercritical) for α < 1 (α > 1). In contrast to the SOC model, here the postsynaptic neurons were assigned randomly at each step. The other parameters were analogous to the SOC model: The model had 2500 neurons with *k* = 4 connections each, and α and *h* were balances such that neurons spiked with *r* = 5 Hz (except if *h* → 0). The open boundary conditions were implemented by defining *p*_*diss*_ = 0.001 as the probability that a neuron projected “outside of the grid,” i.e., the probability that an activation of a postsynaptic neuron was not effective. Note that *p*_*diss*_ > 0 makes the model slightly subcritical. Subsampling was implemented in the same manner as in the SOC model. Note however that spatial distances have no meaning in this model because of its random topology. Results for this model were qualitatively similar to those of the SOC model. Therefore, we usually reported the results of the SOC model only.

### Experiments

We evaluated spikes from recordings in three different species, namely in rats, cats and monkeys. The rat experimental protocols were approved by the Institutional Animal Care and Use Committee of Rutgers University (Mizuseki et al., 2009). The cat experiments were performed in accordance with guidelines established by the Canadian Council for Animal Care (Blanche, 2009). The monkey experiments were performed according to the German Law for the Protection of Experimental Animals, and were all approved by the Regierungspräsidium Darmstadt. The procedures also conformed to the regulations issued by the NIH and the Society for Neuroscience.

The spike recordings from the rats and the cats came from the NSF-founded CRCNS data sharing website (Blanche, 2009; Mizuseki et al., 2009). In brief, in rats the spikes were recorded in CA1 of the right dorsal hippocampus during an open field task. We used the first data set of each animal (ec013.527, ec014.277, ec015.041, ec016.397), and from rat “ec014” we also used a second data set (ec014.333). The five datasets provided sorted spikes, i.e., {37, 77, 32, 58, 58} single units and {4, 8, 8, 8, 8} multi units, respectively. However, since the identity of a unit does not matter for the definition of neural avalanches (see below), the single- and multi-unit activity was combined to one set of spike times. More details on the experimental procedure and the datasets proper can be found on Mizuseki et al. (2009).

For the spikes from the cat, neural data were recorded by Tim Blanche in the laboratory of Nicholas Swindale, University of British Columbia, and downloaded from the NSF-funded CRCNS Data Sharing website (Blanche, 2009). We used the data set pvc3, i.e., recordings in area 18 which contain 50 sorted single units (Blanche and Swindale, 2006). We used that part of the experiment in which no stimuli were presented, i.e., the spikes reflected spontaneous activity in the visual cortex of the anesthetized cat. Details on the experimental procedures and the data proper can be found in Blanche and Swindale (2006); Blanche (2009).

In the monkey experiments, spikes were recorded simultaneously from up to 16 single-ended micro-electrodes (ø = 80 μm) or tetrodes (ø = 96 μm) in lateral prefrontal cortex of three trained macaque monkeys (M1: 6 kg ♀ M2: 12 kg ♂ M3: 8 kg ♀). The electrodes had impedances between 0.2 and 1.2 MΩ at 1 kHz, and were arranged in a square grid with inter electrode distances of either 0.5 or 1.0 mm. The monkeys performed a visual short term memory task with on average 80% correct behavioral responses which required them to memorize a sample object and to compare a test stimulus presented after a delay of 3 s to memory content. The monkeys indicated via differential button press whether test and sample stimuli matched or not. Each trial consisted of a 1 s long baseline, 500–900 ms sample stimulus presentation, a delay of 3 s and a response interval lasting throughout a 2 s test stimulus presentation. More details of the experimental procedure can be found in Pipa et al. (2009). In total, we analyzed spike data from 11 experimental sessions comprising almost 12.000 trials. In M1 and M2 we recorded four sessions each, and in M3 we recorded 3 sessions. 6 out of 11 sessions were recorded with tetrodes (2/4, 4/4, and 0/3 from M1, M2, and M3, respectively). Spike sorting on the tetrode data was performed using a Bayesian optimal template matching approach as described in Franke (2011) (see Franke et al., 2010 for an earlier version) using the “Spyke Viewer” software (Pröpper and Obermayer, 2013). On the single electrode data, spikes were sorted with a multi-dimensional PCA method (Smart Spike Sorter by Nan-Hui Chen).

### Measures

Avalanches in SOC systems are cascades of spikes triggered by a single external spike (Bak et al., 1987). An avalanche can span the entire system, but can also affect just a few sites before it dies out. By definition, in SOC models subsequent avalanches are separated by pauses that are much longer than the avalanches proper (STS) (Bak et al., 1987; Pruessner, 2012). This means that a new avalanche is only triggered after the previous one has long died out. In SOC systems, several avalanche characteristics, such as the distribution of sizes and durations, follow scaling laws, known from the framework of “renormalization theory” (Stanley, 1971, 1999; Sethna et al., 2001; Dhar, 2006). In the following, we define the avalanche measures and describe the expected scaling laws for the SOC model and the critical stochastic branching model.

The avalanche size *s* is the total number of spikes in an avalanche. The avalanche size distribution *f(s)* is its frequency of avalanche sizes, and *p(s)* refers to the respective probability distributions. *f(s)* follows a power law in SOC systems:

$$f\text{​​}\left(s\right)\sim s^{-\tau }$$

*τ* is the critical exponent and depends on the SOC model. For the SOC model we use here (α = 1 and *h* → 0), τ ≈ 1 (Bak et al., 1987; Priesemann et al., 2009), and for the critical branching model τ = 1.5 (Harris, 1963; Haldeman and Beggs, 2005).

The definition of avalanche sizes in the driven models (*h* > 0) and *in vivo* relied on temporal binning (Beggs and Plenz, 2003), since these systems lacked STS. When applying temporal bins to a spike train, the avalanche size was defined as the total number of events in subsequent, non-empty time bins (Figure 1). Stating it differently, an avalanche is by definition the activity in a sequence of full bins, and is preceded and followed by an empty bin. With this definition, *f(s)* changed with the bin size (Figure 1).

As stated above, *f(s)* changed with the bin size. To quantify the bin-size dependent changes of *f(s)*, we used the mean avalanche size (<*s*>), and the measure *f*(*s* = 1, *bs*), i.e., the bin size dependence of the frequency of avalanches of size *s* = 1.

A common measure to characterize neural avalanches is the branching parameter. In a branching process, the branching parameter σ defines whether activity expands (σ > 1) or dies out (σ < 1) (Harris, 1963). Between these two regimes, at σ = 1, the branching process is critical (Harris, 1963). In analogy, the σ^*^ was estimated from spike trains using temporal binning as follows (Beggs and Plenz, 2003; Priesemann et al., 2009): σ^*^_i_ is the number of events in time bin *t*_*i*_ divided by the number of events in time bin *t*_*i* − 1_. The average over all σ^*^_i_ (for which the number of events in *t*_*i* − 1_ is not zero) is defined as the estimated branching parameter σ^*^ (Figure 1) (Beggs and Plenz, 2003; Priesemann et al., 2009). Note that σ^*^ depends on the bin size, and may fail to provide the intended results (see Results and Discussion).

Detrended fluctuations analysis (DFA) (Peng et al., 1994, 1995; Kantelhardt et al., 2002) quantifies long-range correlations in a time-series, which also dominate SOC systems. We applied DFA to the time course of the summed population activity. The summed population activity is the total number of spikes across all neurons at each sampling step. For the DFA, we used analysis window widths from 2^4^ to 2^11^ ms. Smaller window widths could not be used because of the limited sampling resolution, and for windows larger than 2 s the power law scaling broke down, and this impeded the estimation of the DFA exponent β.

It sometimes is helpful to measure the bin size not in absolute time (e.g., milliseconds), but in “average inter event intervals” (<*IEI*>). The <*IEI*> is the inverse of the population rate *R*, i.e., the rate of all units together, independent of their origin. In contrast to the population rate *R*, the rate of a single unit is denoted with *r*.

### LFP recordings in humans

We evaluated LFP which were recorded with intracranial depth recordings in humans. We used the very same data and analysis methods as in Priesemann et al. (2013), and we used the results from all vigilance states combined, because we already showed that the differences with vigilance states were small (Priesemann et al., 2013). We analyzed data from five subjects [3 females (aged 21, 23, and 27), two males; (aged 25 and 48)] with refractory partial epilepsy undergoing pre-surgical evaluation. The subjects were hospitalized between February 2005 and March 2007 in the epilepsy unit at the Pitié-Salpetrière hospital in Paris. All patients gave their informed consent and procedures were approved by the local ethical committee (CCP). Each patient was continuously recorded during several days (duration range: 9–20 days; mean duration: 16 days) with intracranial and scalp electrodes (Nicolet acquisition system, CA, US). Depth electrodes were composed of 4–10 cylindrical contacts (2.3-mm long, 1-mm in diameter, 10-mm apart center-to-center), mounted on a 1 mm wide flexible plastic probe. Pre and post implantation MRI scans were evaluated to anatomically locate each contact along the electrode trajectory. The placement of electrodes within each patient was determined solely by clinical criteria. Signals were digitized at 400 Hz. The five subjects were implanted with (44, 48, 50, 50, and 63) intracranial LFP recording sites. In total seven recording sites were excluded from the analysis due to artifacts and thus we used (44, 48, 45, 50, and 61) recordings sites for data evaluation. All LFP were low-pass filtered at 40 Hz (4th order butterworth, MATLAB) to reduce the impact of line noise.

To analyze the neuronal avalanches for these LFP data in the same manner as the spike data, we extracted binary events from the LFP. These binary events represent phases of enhanced synaptic activity. To extract these events, we calculated the area under the positive deflection lobes between two zero crossings of the LFP (Figure 2 in Priesemann et al., 2013). As LFP-voltages reflect current flows via Ohm’*s* law, this time integral, or area under the voltage curve, is proportional to the total amount of displaced charges and hence describes the departure from equilibrium (charge neutrality) quantitatively—in contrast to simple voltage peaks. To obtain binary events from the LFP, we applied a threshold to the area values under the LFP deflection lobe. The threshold was selected such that each recording site in each interval of constant vigilance state had the same event rate *r* = 1/4 Hz. In contrast to our first paper with these data (Priesemann et al., 2013), we here used only one value for *r*, and combined the results for all vigilance states from wakefulness to deep sleep, since neither *r* nor the different vigilance states affected the results qualitatively (Priesemann et al., 2013).

For the avalanche analysis in the humans, we used a bin size either in units of average inter event intervals (<*IEI*>) or in ms. The <*IEI*> is a function of the event rate *r* and the number of electrode contacts *N*, <*IEI*> = *1/(r · N) = 1/R*. Since *r* was fixed and *N* did not vary much across patients, the following approximation holds: 1 <*IEI*> ≈ 80 ms.

## Funding

Viola Priesemann received financial support from the German Ministry for Education and Research (BMBF) via the Bernstein Center for Computational Neuroscience (BCCN) Göttingen under Grant No. 01GQ1005B. Viola Priesemann and Matthias H. J. Munk received funding from the Federal Ministry of Education and Research (BMBF) Germany under grant number 01GQ0742. Viola Priesemann, Michael Wibral, and Jochen Triesch received funding from the LOEWE Grant “Neuronale Koordination Forschungsschwerpunkt Frankfurt (NeFF).” Robert Pröpper received funding from the Deutsche Forschungsgemeinschaft (GRK 1589/1). Danko Nikolić received funding from the Deutsche Forschungsgemeinschaft (NI 708/5-1) and the Hertie Stiftung. Jochen Triesch is supported by the Quandt foundation.

### Conflict of interest statement

The authors declare that the research was conducted in the absence of any commercial or financial relationships that could be construed as a potential conflict of interest.

## Measures, variables, and abbreviations

α: connection strength or synaptic strength
β: scaling exponent (DFA)
σ: branching parameter
σ^*^: estimated branching parameter
τ: critical exponent of the avalanche size distribution
*bs*: bin size
DFA: detrended fluctuation analysis
*f(s)*: avalanche size distribution
*f*(*s* = 1, *bs*): frequency of avalanches of size *s* = 1 and their dependence on the bin size
*h*: rate of input spikes, also called drive (Hz)
<s>: mean avalanche size
<*IEI*>: average inter event interval; <IEI> = 1/*R*
*N*: number of sampled (model) neurons
*r*: rate per unit (Hz)
*R*: population rate (Hz)
STS: separation of time scales.
