# Supplementary material for: Spike avalanches in vivo suggest a driven, slightly subcritical brain state
Source: Front Syst Neurosci. 2014 Jun 24;8:108. doi: 10.3389/fnsys.2014.00108 (PMC4068003; doi:10.3389/fnsys.2014.00108)
Supplement: Supplementary file 1 [file SupplementaryMaterial.PDF]

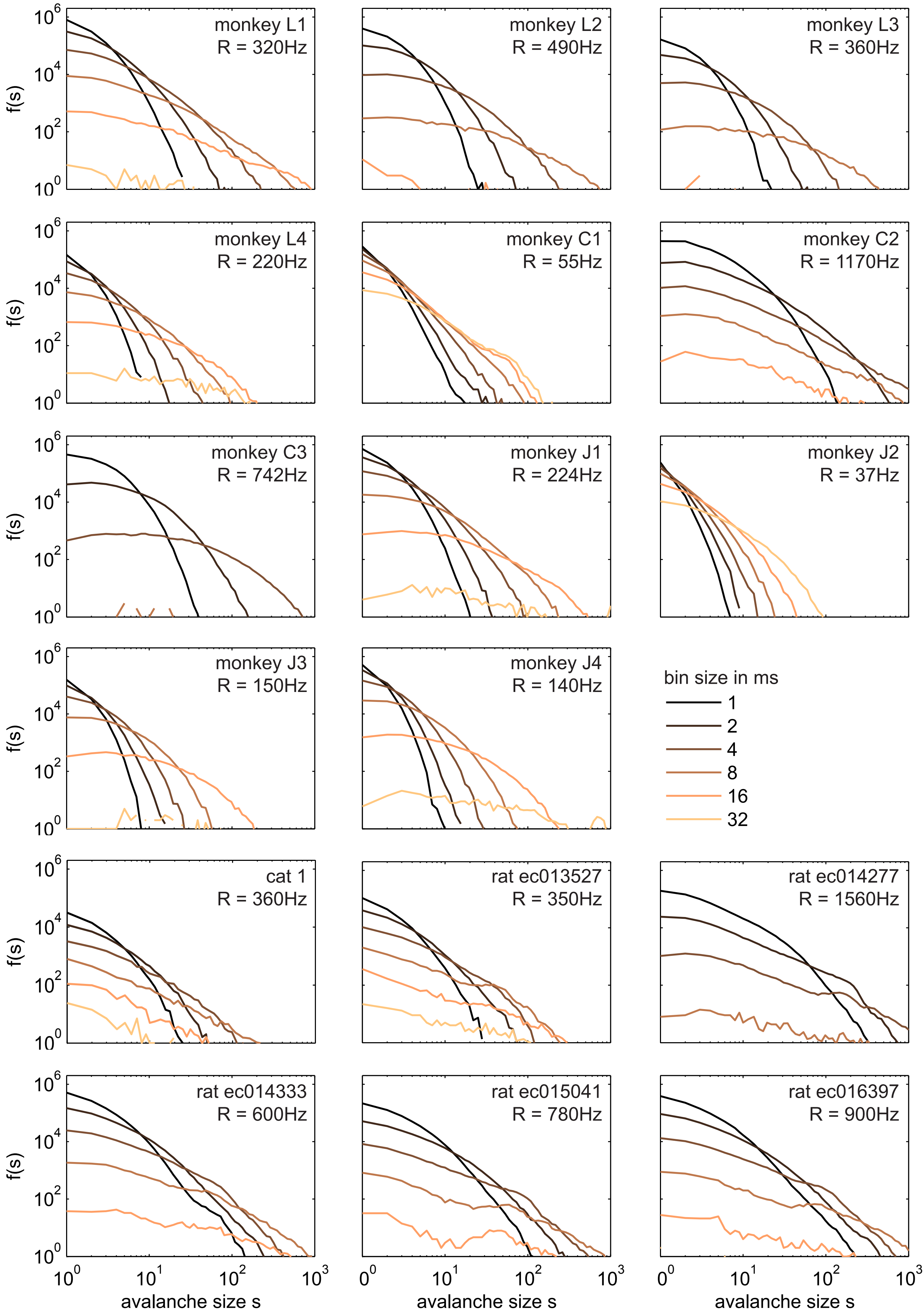

Figure S1

These pannels show the spike *f(s)* for each of the *in vivo* recordings. The experiment ID and the population rate *R* is indicated in each plot. For the rat and cat recordings, the name is the same one used on the CRCNS data sharing platform (Blanche, 2009; Mizuseki et al., 2009), from which we obtained the data.

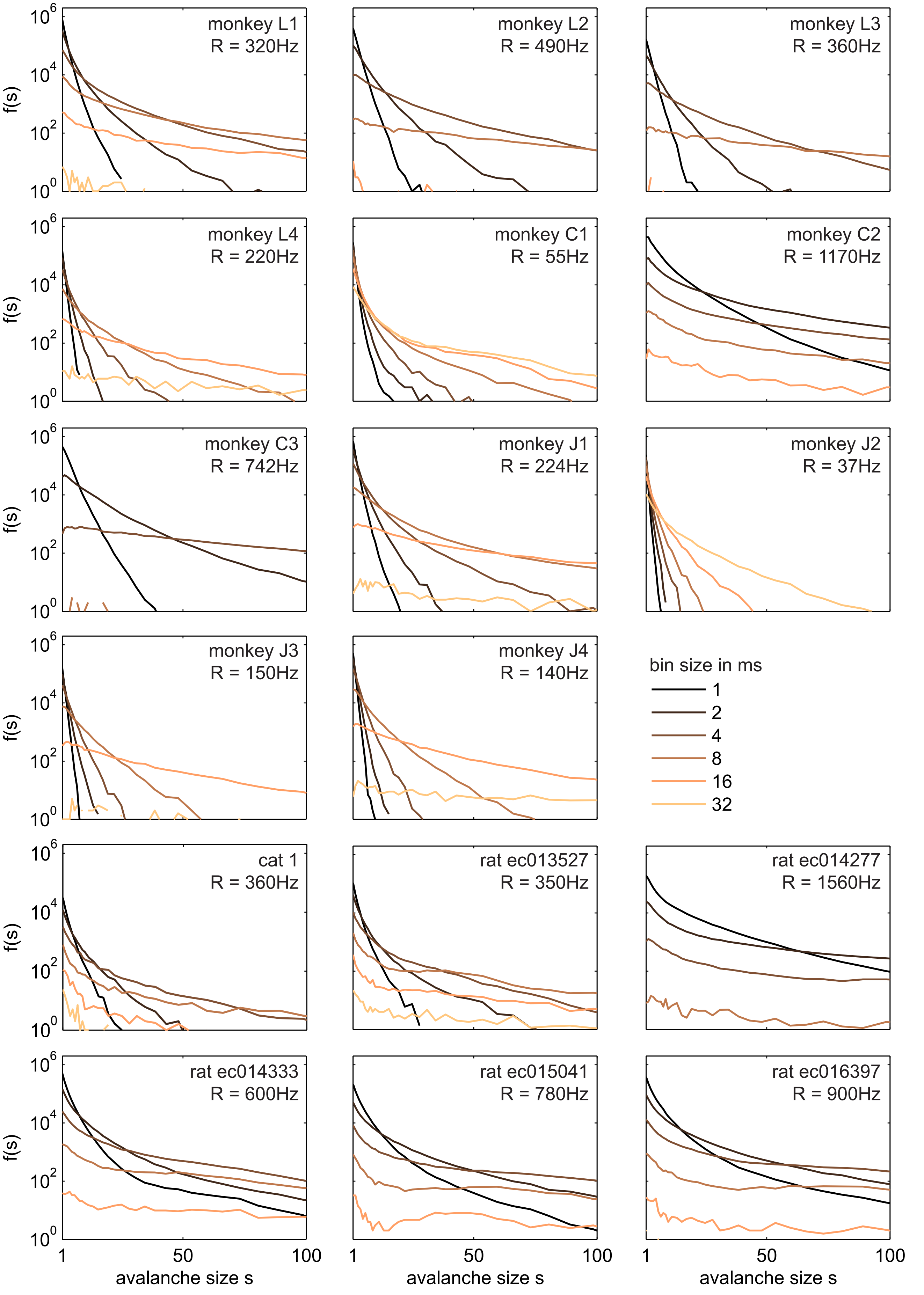

Figure S2

This figure is the same as Figure S1, but with log-linear axes instead of double logarithmic axes. It illustrates that none of the *f(s)* follows an exponential distribution (i.e. a straight line here).

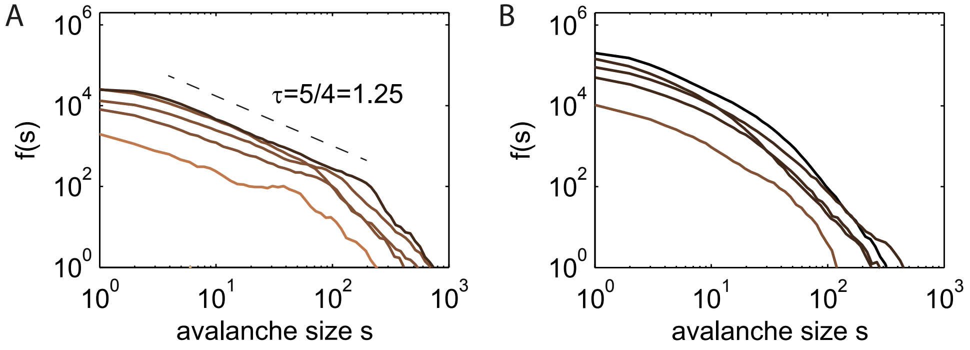

Figure S3

A. For each of the *in vivo* rat recordings, the one avalanche size distributions *f(s)* was selected, which resembled a power law best. The bin sizes of these *f(s)* ranged between 2 ms an 8 ms, corresponding approximately to 3 average inter event intervals (<IEI>). Assuming a power law, the slope would be 1.25 or less (dashed line). **B.** For each of the *f(s)* in A, the corresponding *f(s)* with half the bin size was plotted (approximately 1.5 <IEI>). These *f(s)* clearly deviated from power laws.

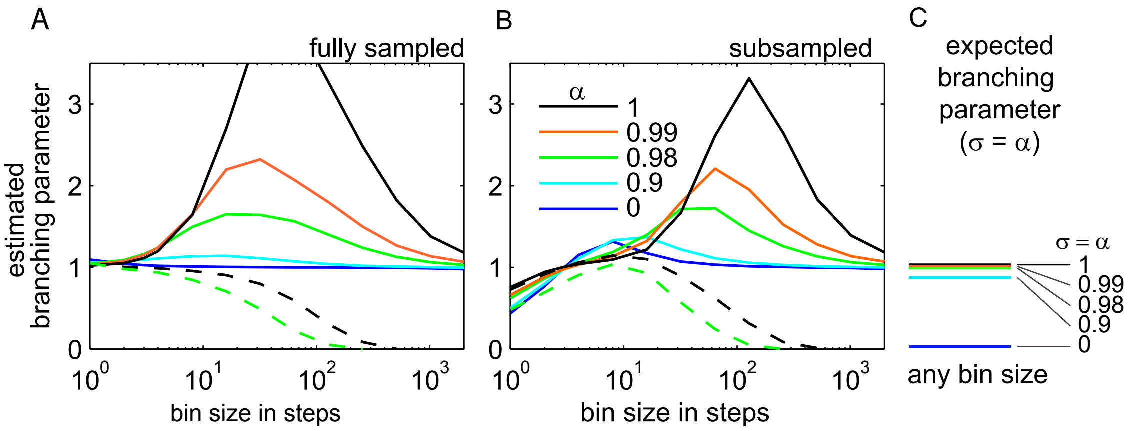

Figure S4

This figure shows the same as Figure 7 in the main text, but for the stochastic branching model. In this model, the branching parameter equals the synaptic strength, i.e. $\sigma = \alpha$. However, the *estimated* branching parameter σ^* differed from α. **A, B.** For the driven models (full lines), the spike rate was fixed to $r = 5$ Hz, while for the model with separation of time scales the drive was infinitesimal small ($h \rightarrow 0$; dashed lines). **A.** Results for the fully sampled model. **B.** Results for the subsampled model ($N = 100$ neurons). **C.** In the stochastic branching model, the branching parameter σ is a model parameter, and is therefore independent of the bin size. Its value is depicted using the same axes as in A, B.
